# Cerebral Semaphorin3D is a novel risk factor for age-associated cognitive impairment

**DOI:** 10.1186/s12964-023-01158-5

**Published:** 2023-06-14

**Authors:** Chien-Yuan Chen, Yung-Mei Chao, Ching-Chang Cho, Cheng-Sheng Chen, Wei-Yong Lin, Yi-Hung Chen, Marlène Cassar, Cecilia S. Lu, Jenq-Lin Yang, Julie Y. H. Chan, Suh-Hang H. Juo

**Affiliations:** 1 Graduate Institute of Medicine, College of Medicine, Kaohsiung Medical University, Kaohsiung, Taiwan; 2 Institute for Translational Research in Biomedicine, Kaohsiung Chang Gung Memorial Hospital, Kaohsiung, Taiwan; 3 Institute of Translational Medicine and New Drug Development, China Medical University, Taichung, Taiwan; 4 Department of Medical Research, China Medical University Hospital, Taichung, Taiwan; 5 Department of Psychiatry, Kaohsiung Medical University Hospital, Kaohsiung, Taiwan; 6 Department of Psychiatry, College of Medicine, Kaohsiung Medical University, Kaohsiung, Taiwan; 7 Graduate Institute of Integrated Medicine, China Medical University, Taichung, Taiwan; 8 Brain Diseases Research Center, China Medical University, Taichung, Taiwan; 9 Graduate Institute of Acupuncture Science, China Medical University, Taichung, Taiwan; 10 Chinese Medicine Research Center, China Medical University, Taichung, Taiwan; 11 Formation and Regulation of Neuronal Connectivity Research Unit, Okinawa Institute of Science and Technology Graduate University, Okinawa, Japan; 12 Institut du Cerveau Et de La Moelle Epinière (ICM)-Sorbonne, UniversitéInserm, CNRS, Hôpital Pitié-Salpêtrière, Paris, France; 13 Graduate Institute of Biomedical Sciences, China Medical University, Taichung, Taiwan; 14 Drug Development Center, China Medical University, Taichung, Taiwan

**Keywords:** Sema3D, Neurodegeneration, Cognition, Aging, Autophagy, miR-195

## Abstract

**Background:**

We previously reported that miR-195 exerts neuroprotection by inhibiting Sema3A and cerebral miR-195 levels decreased with age, both of which urged us to explore the role of miR-195 and miR-195-regulated Sema3 family members in age-associated dementia.

**Methods:**

miR-195a KO mice were used to assess the effect of miR-195 on aging and cognitive functions. Sema3D was predicted as a miR-195 target by TargetScan and then verified by luciferase reporter assay, while effects of Sema3D and miR-195 on neural senescence were assessed by beta-galactosidase and dendritic spine density. Cerebral Sema3D was over-expressed by lentivirus and suppressed by si-RNA, and effects of over-expression of Sema3D and knockdown of miR-195 on cognitive functions were assessed by Morris Water Maze, Y-maze, and open field test. The effect of Sema3D on lifespan was assessed in *Drosophila*. Sema3D inhibitor was developed using homology modeling and virtual screening. One-way and two-way repeated measures ANOVA were applied to assess longitudinal data on mouse cognitive tests.

**Results:**

Cognitive impairment and reduced density of dendritic spine were observed in miR-195a knockout mice. Sema3D was identified to be a direct target of miR-195 and a possible contributor to age-associated neurodegeneration as Sema3D levels showed age-dependent increase in rodent brains. Injection of Sema3D-expressing lentivirus caused significant memory deficits while silencing hippocampal Sema3D improved cognition. Repeated injections of Sema3D-expressing lentivirus to elevate cerebral Sema3D for 10 weeks revealed a time-dependent decline of working memory. More importantly, analysis of the data on the Gene Expression Omnibus database showed that Sema3D levels were significantly higher in dementia patients than normal controls (*p* < 0.001). Over-expression of homolog Sema3D gene in the nervous system of *Drosophila* reduced locomotor activity and lifespan by 25%. Mechanistically, Sema3D might reduce stemness and number of neural stem cells and potentially disrupt neuronal autophagy. Rapamycin restored density of dendritic spines in the hippocampus from mice injected with Sema3D lentivirus. Our novel small molecule increased viability of Sema3D-treated neurons and might improve autophagy efficiency, which suggested Sema3D could be a potential drug target.

Video Abstract

**Conclusion:**

Our results highlight the importance of Sema3D in age-associated dementia. Sema3D could be a novel drug target for dementia treatment.

**Supplementary Information:**

The online version contains supplementary material available at 10.1186/s12964-023-01158-5.

## Background

Aging affects neuronal metabolism, function and survival, all of which contribute to cognitive decline and neurodegenerative diseases [1, 2]. Impairment of hippocampal and surrounding cortical neurons are earlier pathological features of age-related neurological disorders [3, 4]. Furthermore, coordination of hippocampus and neighboring brain regions plays a critical role in cognitive regulation and memory consolidation [5, 6].

Autopsy and molecular studies revealed that abnormal protein metabolism, dendritic spine reduction, and ultimately neuronal death [7, 8] are characteristic features in neurodegenerative diseases such as Alzheimer’s disease (AD) and frontotemporal lobar degeneration (FTLD) [9]. Furthermore, loss of neural stem cells (NSC) leading to reduced neuro-regeneration could exacerbate functional decline in the aging brain. Given the growing size of the aging population and the complexity of neurodegeneration, there is a need for continuous search for key factors governing the brain-aging process.

Our group has previously reported that miR-195 exerts neuroprotection and improves functional recovery in acute stroke rats [10]. Other studies have also demonstrated that miR-195 ameliorates cognitive deficits in early AD [11], and miR-195 alleviates hypoperfusion-induced dementia [12]; accordingly, it is reasonable to postulate that miR-195 might play a role in regulating neuronal functions in the aging brain. Class III Semaphorins (Sema3A-3G) have previously been reported as axon guidance molecules [13]. Both Sema3A and 3D have been shown to stimulate the peripheral branching of axon from soma [14]. Sema3 are secreted proteins that primarily bind to the class A plexin receptors (PlexinA1–PlexinA4) and recent studies have revealed that Sema3 family members are also involved in additional functions related to pathogenesis of several diseases, such as neurodegenerative diseases and diabetic retinopathy [15, 16]. Moreover, we have recently shown that Sema3A is a direct target of miR-195 and partakes in neuron damage in acute stroke [10].

The primary aim of the present study was to elucidate the role of the novel factor Sema3D on cognitive function during the brain aging process. We first demonstrated the protective role of miR-195 in cognitive functions then we identified Sema3D as a direct target of miR-195 and also a key contributor to neurodegeneration and cognitive impairment. Through a series of molecular studies, animal behavior tests, comparisons of lifespan and analysis of data on human gene expression in the brain, we concluded that cerebral Sema3D is a novel risk factor for neurodegeneration and cognitive impairment. Furthermore, we developed a novel small molecule to demonstrate that Sema3D can be a druggable target, which sheds light on new prevention and treatment of cognitive impairment.

## Materials and methods

### Wild-type (WT), miR-195a knockout (KO) mice and aged mice

WT C57BL/6 mice and miR-195a KO mice on a C57BL/6 background were used in this study. Wild-type C57BL/6 mice aged 4, 12, and 21 months were obtained from the National Laboratory Animal Center, Taiwan. Mice were acclimated for at least two weeks before behavioral experiments or being sacrificed. The Animal Care and Use Committee of the China Medical University approved the animal experimental protocols (approval number CMUIACUC-2017–292), which strictly conformed to the Guide for the Care and Use of Laboratory Animals, 8^th^ Edition (2011).

### Animal studies

First, miR-195a KO and WT mice were used to investigate the role of miR-195 in cognitive functions, neurogenesis, and brain senescence, with operators being blinded to the genotype during testing. Cognitive functions of miR-195a KO mice and WT were assessed by the Morris Water Maze (MWM) test, Y-maze test and open field test (OFT). Neurogenesis ability was evaluated by quantifying SOX2 positive neural stem cells (NSCs) in the dentate gyrus (DG) and subventricular zone (SVZ). The senescence-associated β-galactosidase (SA-β-gal) activity and p16^INK4a^/p19^Arf^ expression were used to assess brain senescence. To explore lifespan of miR-195a KO mice, we used the historical life-span data on wildtype C57BL/6 mice to compare with that in miR-195a KO mice due to the limited space and resources, so the magnitude of life-span reduction needs to be interpreted with caution [17].

Secondly, we explored the role of Sema3D in cognitive functions, neurodegeneration and neurogenesis using Sema3D-overexpressing mice with operators again being blinded to the treatment of the mice during testing. Cognitive functions were assessed by the MWM test, novel object recognition test, Y-maze test, and OFT. To support our findings in behavioral tests, we used the Golgi-cox stain to detect neurodegeneration in the same mice after testing. If the Golgi-cox stain showed a decline of dendritic spine density of neurons, there was neurodegeneration in the brain. To explore the effect of Sema3D on autophagy, the hippocampus was collected from the same mice subjected to behavioral tests, and autophagy-related proteins were analyzed using western blot. To investigate the effect of Sema3D on neurogenesis, recombinant Sema3D protein was bilaterally intracerebroventricularly (ICV) injected into 4-month-old WT mice. Neurogenesis ability was evaluated by quantifying SOX2-positive NSCs in the dentate gyrus (DG) and subventricular zone (SVZ).

Two in vivo rescue studies were conducted to confirm the detrimental effect of Sema3D. Firstly, a single dose of Rapamycin (0.2 nmol/0.2 µL/injection site) and Lv.Sema3D was injected to the bilateral hippocampi. Golgi-cox stain to reveal dendritic spine density was performed to evaluate the severity of Sema3D-induced neurodegeneration. Secondly, Sema3D siRNA or control siRNA was delivered into the hippocampus of 12-month-old miR-195a KO mice. Spatial working memory and locomotor function acquired by the Y-maze tests were used to evaluate the effect of Sema3D siRNA on cognitive functions. Again, the Golgi-cox stain was used to assess the rescue effect. Stereotactic coordinates for all experiments in the present study are shown in Supplementary Table S3. Operators were blinded to genotype and treatments.

### Cell culture and cell studies

Human neuron cell line SY5Y (ATCC CRL-2266) were obtained from American Type Culture Collection, USA. HEK293 cells (BCRC90016) were obtained from Bioresource Collection and Research Center, Taiwan. SY5Y cells and HEK293 cells were maintained in DMEM supplemented with 10% FBS (Invitrogen, Waltham, MA, USA), 1% penicillin and streptomycin (Biowest, Loire Valley, France), and 1% L-glutamine (Invitrogen) in a humidified incubator under an atmosphere of 5% CO_2_ at 37 °C. Human neural stem cells (NSCs) were induced from human iPSCs. In brief, human iPSCs were first cultured as embryoid bodies (EBs) in EB medium on Matrigel (BD Biosciences; Franklin Lakes, NJ, USA)‐coated dishes and supplemented with recombinant Noggin protein (250 ng/ml, R&D). On day 10, medium was replaced with EB medium supplemented with Sonic Hedgehog (SHH, 20 ng/ml, R&D) and fibroblast growth factor 8 (100 ng/ml, R&D). Upon the appearance of rosette‐like structures (day 14), medium was changed to medium supplemented with BDNF, ascorbic acid, SHH and FGF8. On day 22, FGF8 was withdrawn, and cells were maintained in the medium supplemented with BDNF, ascorbic acid and SHH. On day 29, cells were seeded on poly‐L‐ornithine/laminin‐coated dishes in complete StemPro NSC Medium and were then expanded up to 10 passages.

#### Sema3D and Neurodegeneration

To investigate whether Sema3D induced neurodegeneration via the PI3K/Akt/mTOR/autophagy pathway, SY5Y cells were treated with recombinant Sema3D protein for 72 h and cell lysates were collected for western blot analysis. For the rescue assay, 1 μM Rapamycin and Sema3D were co-administrated into cultured SY5Y cells for 72 h and cell viability was detected by Ki67 staining at 72 h. CHOV20191024, the novel Sema3D antagonist, and Sema3D were co-treated with human neuron SY5Y to demonstrate the bioactivity of CHOV20191024. MTT assay, western blot and cell survival analysis were used to evaluate the rescue ability of CHOV20191024.

#### Sema3D Affects NSC

Sema3D was added to the culture medium of human NSCs. We assessed whether Sema3D could affect NSC stemness by performing sphere formation assay with the number of spheres calculated on Day 5 post-Sema3D treatment.

### Selection and analysis of gene expression datasets

The Gene Expression Omnibus (GEO) database (as of December 2019) was queried for human microarray gene expression datasets in response to neurodegenerative diseases and aging. We first focused on gene expression data on the hippocampus (Table 1) and then on the cortex & cerebellum (Table 2). For the hippocampus data, the specific search terms used were: “neurodegeneration”, “dementia”, “cognitive impairment” and “postmortem brain”, with the retrieved datasets being filtered based on the following criteria: 1) raw data available and derived from human hippocampal tissue; 2) for neurodegenerative disease datasets, at least one control group (normal subjects) and one diseased group; and 3) *Sema3D* should be detected in the microarray results. Table S1 summarizes all retrieved datasets along with the reasons for their inclusion or exclusion from our analyses.

**Table 1:** List of hippocampal Sema3D and Sema3A expression levels in neurodegenerative diseases and aging datasets. For each dataset, all subjects were classified according to the severity and phenotypes provided by the original article. The expression levels in all severity groups were normalized to the data of normal subjects in each correspondent dataset. MMSE: Mini-Mental State Examination. Data are presented as mean ± SEM. *p < 0.05, **p < 0.01 using Student's *t*-test. # *p* = 0.080, ## *p* = 0.1658 using one-way ANOVA

**Datasets number**	**Phenotype**		**Fold change of Sema3 (Phenotype Severity: Low → High)**			
GSE11882	Normal Aging		age = 20 ~ 49 *n* = 16	age = 50 ~ 79 *n* = 10	age = 80 ~ 100 *n* = 16	
		**Sema3D**	**1 ± 0.04**	**1.14 ± 0.18**	**2.49 ± 1.19**	
		Sema3A	1 ± 0.004	1.00 ± 0.01	0.91 ± 0.03	
GSE1297	Alzheimer's Disease (AD)		Normal (Mean age = 85.33 yr; *n* = 9)	Incipient (Mean age = 91.86 yr; *n* = 6)	Moderate (Mean age = 83.38 yr; *n* = 8)	Severe (Mean age = 84.00 yr; *n* = 7)
		**Sema3D**	**1 ± 0.18**	**0.86 ± 0.11**	**1.47 ± 0.31**	2.16 ± 0.60 *
		Sema3A	1 ± 0.07	1.10 ± 0.25	1.14 ± 0.09	1.26 ± 0.22
GSE48350	Alzheimer's Disease (AD)		Normal (Mean age = 62.49 yr, *n* = 43)	AD (Mean = 83.05 yr, *n* = 19)		
		**Sema3D**	**1 ± 0.06**	1.57 ± 0.27**		
		Sema3A	1 ± 0.02	1.09 ± 0.02 *		
GSE36980	Alzheimer's Disease (AD)		Normal (Mean age = 77.00 yr, *n* = 10)	AD (Mean age = 92.86 yr, *n* = 7)		
		**Sema3D**	**1 ± 0.10**	**0.85 ± 0.12**		
		Sema3A	1 ± 0.06	0.99 ± 0.10		
GSE84422	Clinical Dementia Rating (CDR)		CDR = 0, normal (Mean age = 85.56 yr, *n* = 9)	CDR = 0.5 ~ 1 (Mean age = 84.58 yr, *n* = 19)	CDR = 2 ~ 3 (Mean age = 89.41 yr, *n* = 17)	CDR = 4 ~ 5 (Mean age = 85.20 yr, *n* = 10)
		**Sema3D**	**1 ± 0.01**	**0.97 ± 0.01**	**1.07 ± 0.03**	1.14 ± 0.03**
		Sema3A	1 ± 0.04	0.99 ± 0.03	0.97 ± 0.03	1.00 ± 0.03
GSE13162	FTLD-U		Normal (Median age = 67; *n* = 2)	Moderate (Median age = 71; *n* = 8)	Severe (Median age = 64; *n* = 5)	
		**Sema3D**	**1 ± 0.03**	**1.08 ± 0.06**	**1.31 ± 0.11 #**	
		Sema3A	1 ± 0.29	0.96 ± 0.06	1.26 ± 0.14 ##

**Table 2:** Sema3D expression levels of the cortex and cerebellum in human Alzheimer's disease datasets. For each dataset, all subjects were classified according to the severity and phenotypes provided by the original article. The expression levels in all severity groups were normalized to the data of normal subjects in each correspondent dataset. CDR: clinical dementia rating; NFT: neurofibrillary tangles. Data are presented as mean ± SEM. *p < 0.05, **p < 0.01, ***p < 0.001 using Student's *t*-test

Datasets number	Phenotype	Tissue	Fold change of Sema3D (Severity: Low → High)			
GSE33000	Alzheimer's Disease (AD)	Prefrontal cortex	**1.00 ± 0.04** (normal, mean age = 68.6; *n* = 104)	1.81 ± 0.06*** (Young AD, mean age = 68.6; *n* = 70)	2.07 ± 0.07*** (Old AD, mean age = 86.7; *n* = 206)	
GSE44770	Alzheimer's Disease (AD)	Prefrontal cortex	**1.00 ± 0.03** (normal, mean age = 62.6; *n* = 62)	2.01 ± 0.08*** (Young AD, mean age = 62.6; *n* = 14)	2.28 ± 0.08*** (Old AD, mean age = 82.99; *n* = 94)	
GSE44768	Alzheimer's Disease (AD)	Cerebellum	**1 ± 0.04** (normal; *n* = 81)	1.35 ± 0.06*** (AD; *n* = 108)		
GSE44771	Alzheimer's Disease (AD)	Visual cortex	**1 ± 0.05** (normal; *n* = 76)	2.05 ± 0.08*** (AD; *n* = 119)		
GSE185909	Alzheimer's Disease (AD)	Prefrontal cortex	**1 ± 0.97** (normal; *n* = 7)	**1.13 ± 1.17** (MCI; *n* = 10)	**1.41 ± 0.85** (AD; *n* = 17)	
GSE131617	Alzheimer's Disease (AD)	frontal cortex	**1.00 ± 0.39** (NFT 0, normal; *n* = 13)	**1.04 ± 0.56** (NFT I-II; *n* = 20)	**1.03 ± 0.48** (NFT III-VI; *n* = 19)	**1.03 ± 0.47** (NFT V-VI; *n* = 19)
GSE84422	Alzheimer's Disease (AD)	frontal cortex	**1.00 ± 0.15** (CDR = 0, normal; *n* = 11)	**0.99 ± 0.13** (CDR = 0.5 ~ 1; *n* = 17)	**1.03 ± 0.22** (CDR = 2 ~ 3; *n* = 23)	**1.03 ± 0.47** (NFT V-VI; *n* = 19)

Raw gene expression data and disease severity classification were obtained from the GEO databases. After filtering, six human hippocampal microarray datasets (Table 1) from dementia, aging, frontotemporal lobar degeneration with ubiquitinated inclusions (FTLD-U) and Alzheimer’s disease (AD) were retained for further analysis.

For each transcriptome dataset, raw expression data on *Sema3D* and *Sema3A* were examined and transformed to signal intensity by log2 methods. Next, results of signal intensity were normalized by the average intensity of the control subjects, and classified according to the severity of disease provided by the original article. Lastly, the fold change of Sema3D/Sema3A expression were calculated by comparing the signal intensity between diseased and control groups.

For the *Sema3D* expression data on cortex and cerebellum, the retrieved datasets were filtered based on the following criteria: 1) raw data available and derived from cortex and cerebellum tissue; 2) Alzheimer’s disease dataset; 3) at least 25 dementia subjects; and 4) detection of *Sema3D* in the microarray results. Table S2 summarizes all retrieved datasets along with the reasons for their inclusion or exclusion from our analyses. After filtering, four microarray datasets from Alzheimer’s disease (AD) were retained for further analysis (Table 2). Lastly, transcriptome dataset analysis and fold change of *Sema3D* calculations were performed as mentioned above.

### Sema2A-overexpressing *Drosophila* lifespan and locomotor analysis

Flies carrying *elav-Gal4(X)* (BDSC stock number 458) or *UAS-Sema2a-GFP* (BDSC stock number 65747) were obtained from Bloomington *Drosophila* stock center (BDSC; Indiana University, Bloomington, IN, USA) and grown on a standard cornmeal medium at 25 °C on a 12-h light–dark cycle at 60% relative humidity. To overexpress Sema2A in the nervous system, virgin female flies carrying the neuron-specific driver *elav-Gal4(X)* were crossed to male flies carrying *UAS-Sema2a-GFP*, and their F1 offspring were Sema2A-overexpressing flies, while the F1 offspring of virgin female flies carrying *elav-Gal4(X)* and male wildtype flies were used as control flies.

To investigate the effect of Sema2A on lifespan, Sema2A-overexpressing flies and control flies were used (*n* = 300/group) and flies surviving being calculated every 7 days with the number of dead flies documented and survival curves then graphed. The Gehan-Breslow-Wilcoxon test was used to determine statistical differences between Sema2A-overexpressing flies and control flies.

The locomotor activity of Sema2A-overexpressing flies was determined using the negative Geotaxis assay [18]. Briefly, groups of about 15 flies were placed in a vertical column (25 cm long, 1.5 cm diameter) with a conic bottom end. Upon gently tapping down, flies were startled and responded by climbing up. After 30 s, those flies having reached halfway to the top were counted and the climbing score was determined by the calculated ratio between flies above the midline over the total number of flies, then the Mann–Whitney test was used to determine statistical differences between Sema2A-overexpressing and control flies (*n* = 150/group).

### Template identification and protein homology modeling

The structure of human Sema3D was not available in the RCSB Protein Data Bank (http://www.rcsb.org/), so BLASTP was used to identify homologs in this Databank. The X-ray crystal structure of Sema3A from Mus musculus (PDB code is 4GZ8), which has 60.04% sequence identity to Sema3D, was used as the template during the SWISS-MODEL tool [19, 20]. Optimization of the predicted model of Sema3D was confirmed via VERIFY 3D and PROCHECK programs available in the Structural Analysis and Verification Server (SAVES) (http://nihserver.mbi.ucla.edu/SAVES), where the VERIFY 3D program examined the compatibility of a protein 3D model with any amino acid sequence (1D), and the PROCHECK program evaluated the stereochemical quality of the protein secondary structure [21].

### Structure-based virtual screening using molecular docking

The ZINC database contains commercially available molecules for virtual screening [22] to identify potential molecules against a specific protein target. Xanthofulvin is a Sema3A inhibitor, and analogs with a minimum of 75% similarity to xanthofulvin were selected from the ZINC database with a total of 950 compounds meeting this criterion and being downloaded as our ligand library.

Xanthofulvin has been reported to interfere with the binding between Sema3A and PlexinA receptor [23]. Among all the possible interactions between Sema3 and PlexinA family members, only Sema3A-PlexinA2 complex has been revealed to possess the x-ray structure (PDB code: 4GZA) [19]. Based on the vital residues in the interface of Sema3A-PlexinA2 complex, we predicted the docking site on Sema3D, and to validate this, virtual screening was firstly performed by docking the compound from our ligand library to the binding site of Sema3D using iGEMDOCK software [24] to rank ligands, with the docking program being run at accurate docking (very slow) option (population size (*n* = 800), generations (g = 80) and number of solutions (s = 10)) respectively, to predict the interaction between the ligands and the binding site of Sema3D. For this program, in iGEMDOCK scoring function option, the scoring function of GEMDOCK along with ligand hydrophobic, electrostatic preference and intra-energy (both was set to 1) were all chosen.

The best conformation with the lowest binding energy was selected from the docking screening results. The interactions of Sema3D-ligand complex conformations including bond lengths and hydrogen bonds were analyzed using Pymol and Swiss-PdbViewer v4.0 software [25]. We expected that the virtually screened compound could block Sema3D signaling transduction.

### Statistical analysis

Statistical differences were evaluated using Student’s *t*-test for cross-sectional studies. Data on animal studies with repeated measures were first analyzed by “one-way repeated measures ANOVA” to assess the overall change of cognitive function over time for each type of mice. Then “two-way repeated measures ANOVA” was used to compare the overall difference of cognitive change between two types of animals (such as animals with or without over-expression of cerebral Sema3D). The advantage of using repeated measures ANOVA is to model the correlation between the repeated measures of the same animal. The Gehan-Breslow-Wilcoxon test was used for *Drosophila* lifespan analysis. A *p* value less than 0.05 was considered statistically significant. Analysis of the data and plotting of the figures were performed using Prism 7 (GraphPad Software Inc., CA, USA).

## Results

### Low miR-195 level leads to cognitive impairment

We created a general miR-195a knockout (KO) mouse. Notably, a mouse has two miR-195 genes (miR-195a and miR-195b) and the present study used the mice with miR-195a gene KO because of the following three reasons: firstly, the BLAST search of DNA sequence showed a similarity of 96.3% between mouse miR-195a gene and human miR-195 gene but “no significant similarity” between mouse miR-195b gene and human miR-195 gene; secondly, the RNA sequence of mature human miR-195 and mouse miR-195a is 100% identical, although the RNA sequence of mature mouse miR-195b is different from that of mature human miR-195 by 4 nucleotides (mmu-miR-195b: 5’- uagcagcacagaaauaguagaa-3’; hsa-miR-195: 5’-uagcagcacagaaauauuggc-3’); and thirdly, phenotypically miR-195b KO mice have recurrent skin infections and a low reproductive rate, which implied miR-195b KO might lead to more systemic effects than miR-195a KO. Given the above reasons, miR-195a KO mice was the better model to investigate the correlation between miR-195 and cognitive dysfunctions.

Our previous data revealed that miR-195 levels were reduced by 25%-50% in the brain and 50%–75% in other organs of our miR-195a KO mice [26]; moreover, miR-195 levels are more abundant in the brain than in several internal organs [27] and cerebral miR-195 level decreased with 1age in WT mice [26].

To investigate whether low miR-195 levels could affect cognitive functions, we compared learning, memory, and locomotor function between miR-195a KO and WT mice across different ages. Spatial learning and memory were assessed by the Morris Water Maze (MWM) test carried out according to the scheme depicted in Figure S1A, working memory was measured by the Y-maze test, and locomotor activity was evaluated by the open field test (OFT).

**Fig. 1:**
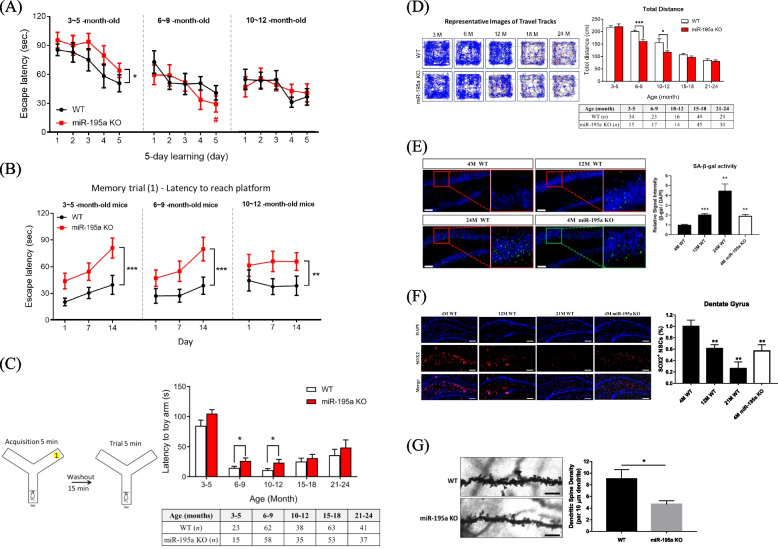
miR-195a KO mice present cognitive impairments and neurodegenerative features. **A**-**B** Learning and spatial memory were evaluated by the Morris Water Maze test (MWM) in miR-195a KO and age-matched WT mice. **A** Longer escape latency to reach the hidden platform indicates lower learning ability. Significant results from one-way repeated measures ANOVA for each type of mice are indicated by pound signs (#), and from two-way repeated measures ANOVA for comparison between KO and WT mice indicated by asterisk signs (*). **B** Longer escape latency to reach the platform indicates worse memory. Two-way repeated measures ANOVA yielded that KO had worse memory than age-matched WT mice. **C** The Y-maze test assesses working memory by measuring the time to reach the arm of the novel toy. Quantitative data is in the right panel. **D** Locomotor function was measured by the open field test. The representative images (left panel) show the traveled tracks of WT and miR-195a KO mice in the open field. The quantitative data are in the right panel. **E** Senescence-associated ß-galactosidase (SA-ß-gal) stain in the hippocampus. The representative images in the left panel show hippocampal senescent cells (green). Scale bar = 100 μm (*n* = 3/each group). **F** miR-195a KO mice had reduced NSC population than did age-matched WT mice. Representative images show SOX2 + (red) NSCs in the hippocampal dentate gyrus (DG) of WT mice and miR-195a KO mice (*n* = 3/group). Magnification: 20X. Scale bar = 200 μm. **G** Decreased dendritic spine density in miR-195a KO mice. Representative images of apical dendritic shaft of CA1 pyramidal neurons from 4-month-old miR-195a KO mice and age-matched WT mice (*n* = 3 mice/group; 15 neurons/mice). Scale bar = 5 μm. Figure 1A: #*p* < 0.05 verse first-day escape latency of the same mice type. *p < 0.05, **p < 0.01, *** p < 0.001

Repeated measure ANOVA was used to assess the sequential changes of cognitive functions within and between mice types (see Statistical Analysis section for details). For spatial learning, the performance of miR-195a KO mice aged 3—5 months was inferior to that of age-matched WT mice (*p* < 0.05), as evidenced by longer averaged time to find the hidden platform and a flat slope in learning curve (Fig. 1A); however, there was no difference in spatial learning between these two groups after age of 5 months (Fig. 1A; data on mice aged 15 ~ 24 months are in Figure S1B). Three parameters assessed memory, two from the MWM test and one from the Y-Maze test (Supplementary Table S1), with results indicating that before age of 12 months, miR-195a KO mice had worse memory than age-matched WT mice in the MWM test (Fig. 1B and Figure S1C) and in the Y-Maze test (Fig. 1C). Another memory parameter in the MWM test was the entry frequency in the target quadrant, which did not reveal any difference between KO and WT mice regardless of age (Figure S1D). miR-195a KO mice had worse locomotor activity compared to WT mice between age of 6 – 12 months (Fig. 1D). Similar to the memory and spatial learning, old KO and WT mice showed no difference in locomotor activity. The above results suggested that general miR-195a KO might induce accelerated aging processes in the brain and locomotor activity in mice.

### Low miR-195 level accelerates premature aging and exacerbates neurodegeneration

Since cognitive dysfunction is one important aging characteristic, we speculated whether miR-195 deficiency was also associated with other aging phenotypes including lifespan, molecular biomarkers, neural stem cells (NSCs) and dendritic spines. miR-195a KO mice had shorter mean and median lifespans by approximately 25% compared to WT mice [17] (Figure S1E). Furthermore, we examined two aging biomarkers in the brain, senescence-associated β-galactosidase (SA β-gal) activity and p16^Ink4a^ / p19^Arf^ expression, using two different SA β-gal staining methods with both showing robust increase in SA β-gal activity observed as early as 4 months of age in the hippocampus and cortex of miR-195a KO mice. In contrast, SA β-gal activity was not increased until 12 months of age in the WT mice (Fig. 1E and Figure S1F). In addition, our SA β-gal staining data on WT mice were consistent with previous reports [28], showing that expressions of p16^Ink4a^ and p19^Arf^ in the brain were significantly elevated in miR-195a KO mice aged 4 months when compared to WT mice (Figure S1G).

A decreased number of NSCs and a low density of dendritic spines in the neurons are other features in the aging brain. We compared the number of SOX2-positive NSCs in the dentate gyrus (DG) and subventricular zone (SVZ) between miR-195a KO and WT mice and as expected, a decrease in the number of NSCs with age in both DG (Fig. 1F) and SVZ (Figure S1H) was found in WT mice. However, a decrease in the number of NSCs by 40% and 50% was found in 4-month-old miR-195a KO mice in both DG and SVZ respectively, which was similar to what was observed in the 12-month-old WT mice. Moreover, histological analysis by Golgi-cox stain revealed that miR-195a KO mice had noticeably lower dendritic spine density by 50% in hippocampal neurons than age-matched WT mice (Fig. 1G).

All of the above experiments consistently indicated that a low level of miR-195 might promote brain aging and impaired cognitive functions, which inspired us to search for the key miR-195-regulated molecules that are engaged in facilitating the brain-aging process.

### Regulation of Semaphorin 3 in the brain by miR-195

We have previously reported that Sema3A is a direct target of miR-195 and over-expression of Sema3A via stressed neurons promoting cell apoptosis [10]. To explore if other Sema3 members were involved in the brain-aging process of miR-195a KO mice, bio-informatics algorithms (miRanda [29] and TargetScan [30]) were used to predict miR-195 target genes in the Sema3 family with Sema3A and Sema3D being revealed as direct targets of miR-195. The luciferase reporter assay confirmed that miR-195 directly binds to *Sema3D* RNA 3'-UTR (Figure S2A). Both Sema3A and Sema3D levels were increased in the hippocampus of miR-195a KO mice (Figure S2B-C) and Sema3D mRNA level was 50% higher than Sema3A in the human hippocampus as revealed by RNA-seq analysis from the Allen Brain Atlas (Figure S2D). Since Sema3A appears associated with neurodegenerative disorders while the role of Sema3D in brain function remains unclear, we decided to further investigate the role of Sema3D in this regard.

### Sema3D level is associated with aging features and neurodegenerative diseases

**Fig. 2:**
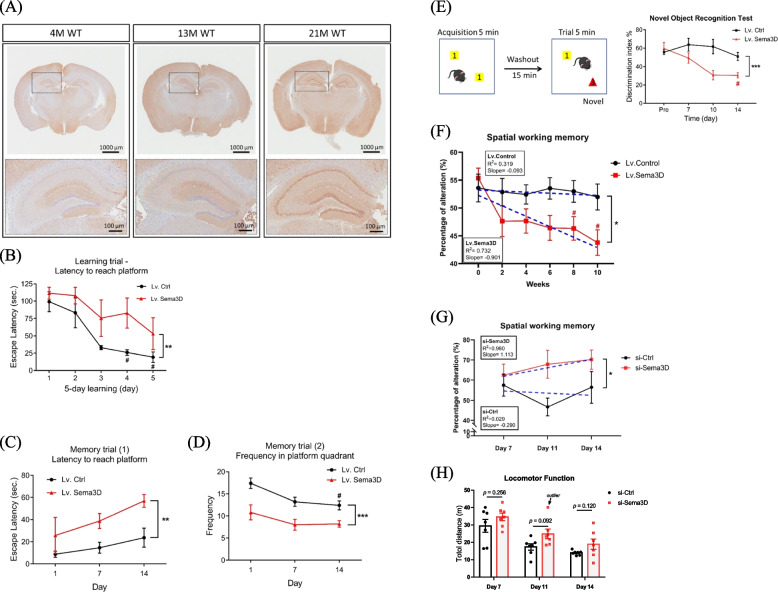
Increased Sema3D levels deteriorate cognitive functions. **A** Representative IHC stain of endogenous Sema3D protein in the brain of WT mice. Upper panel: whole-brain section; Lower panel: hippocampus. **B**-**D** Learning and spatial memory performance were examined by the MWM in control and Sema3D-overexpressing mice aged 4 months (*n* = 4 /group). Significant results from one-way repeated measures ANOVA for each type of mice as indicated by pound signs (#), and from two-way repeated measures ANOVA for comparisons between Sema3D-overexpressing and control mice as indicated by asterisk signs (*). **B** The control mice significantly improved their learning after 4-day practices but Sema3D-overexpressing mice failed to show significant improvement after 5-day practices. **C** Escape latency and **D** frequency were measured in the memory trials. These two types of mice had significant differences in memory over the 14-day test. **E** Sema3D-overexpressing mice significantly lost short-term memory on day 14 in the novel object recognition test (*n* = 4/group). **F** The Y-maze test was performed to measure spatial working memory in mice for 10 weeks (*n* = 14/each group). A lower percentage of alteration indicates worse spatial working memory. Data in Fig. 2B-F are presented as mean ± SEM. **p < 0.01 and ***p < 0.001. #*p* < 0.05 **G** The Y-maze test measuring spatial working memory was used to assess the effect of siRNA-Ctrl or siRNA-Sema3D injected into miR-195a KO mice aged 12 months (*n* = 7/each group). The tests for siRNA-Sema3D and siRNA-Ctrl mice were conducted on the same experiment day. Two-way repeated measures ANOVA showed siRNA-Sema3D significantly improved spatial memory (*p < 0.05). **H** Locomotor function was determined using the total travel distance, with longer distance indicating better locomotor function

To explore the correlation between Sema3D and brain aging, cerebral Sema3D expression was detected in WT mice aged 4, 13, and 21 months. Histological staining revealed an age-dependent increase of Sema3D (Fig. 2A); then, we tested for potential correlation between the brain Sema3D level and dementia using the NCBI Gene Expression Omnibus (GEO) database. Although the hippocampus is often damaged in the early stage of dementia, other brain regions also become involved as the disease progresses; accordingly, we analyzed Sema3D levels in both hippocampus and neighboring brain regions.

Firstly, we analyzed hippocampal Sema3D expression in patients with cognitive impairment, and based on the filtering criteria described in the Methods section and Figure S3A, there were six available GEO datasets containing human hippocampal gene expression profiles with data on Sema3D expression (Supplementary Table S2). These six datasets included one for normal aging, one for dementia according to Clinical Dementia Rating (CDR), three for Alzheimer’s disease (AD), and one for frontotemporal lobar degeneration with ubiquitin-positive inclusions (FTLD-U) (Table 1). Additionally, Sema3A expression was also analyzed as presented in Table 1. Hippocampal Sema3D levels had an age-dependent increase in normal subjects (GSE11882, Table 1), which is consistent with the finding from our rodent brain samples (Fig. 2A). Among the three AD datasets, hippocampal Sema3D levels were significantly higher in the subjects with more severe AD in two datasets (GSE1297 and GSE48350) (Table 1) while also being higher in subjects with more severe clinical dementia (GSE84422) and FTLD-U (GSE13162) (Table 1). Taken together, these data indicated that a high Sema3D level is a common characteristic in dementia patients and also in normal aging subjects, which suggest Sema3D as a contributor to the development of dementia and brain aging.

Furthermore, we analyzed Sema3D levels in the cortex and cerebellum. In the GEO database, there are more data on AD than other neurodegenerative diseases. To exclude less reliable studies, we arbitrarily used at least 25 dementia subjects in a study as a selection criterion. According to the filtering criteria described in Figure S3B, 7 GEO datasets were filtered for Sema3D analysis (Supplementary Table S2). Among these 7 datasets, three studies had data on the prefrontal cortex, two of which showed a significantly higher Sema3D level in the AD patients (*p* < 0.001) and one smaller study yielded an odd ratio of 1.41 without reaching significance level of 0.05 (Table 2). Only one dataset had Sema3D in the cerebellum and one in the visual cortex, both of which included more than 100 AD samples and showed a significantly higher Sema3D level in AD patients (*p* < 0.001 for both datasets). The data on the frontal cortex were available in the remaining two datasets, but AD patients did not have higher Sema3D level in this region. Collectively, these results provided another line of evidence supporting the association between Sema3D and dementia.

### Sema3D is a direct cause of neurodegeneration and cognitive impairment

To establish the causal relationship between cerebral Sema3D protein and neurodegeneration as well as cognitive dysfunctions, Sema3D-expressing lentivirus (Lv.Sema3D) was injected into bilateral hippocampi of 4-month-old WT mice with efficiency of virus transfection confirmed by measuring the level of Sema3D protein on Day 7. Immunoblot data revealed an increase of Sema3D level by threefold in the hippocampus while the Sema3D level in the cortex was only slightly increased (Figure S3C). Histological analysis by Golgi-cox stain revealed a significant decline of dendritic spine density in the hippocampus of Sema3D-overexpressing mice (Figure S3D), which supported a negative effect of Sema3D on the morphology of neuronal cells.

Subsequently, four tests were performed to examine the detrimental effect of over-expression of hippocampal Sema3D on cognitive functions in mice. In addition to the same three tests conducted in miR-195a KO mice, an additional recognition memory examination was also performed using the Novel Object Recognition test (Supplementary Table S1). The scheme of the MWM test is depicted in Figure S3E. Sema3D-overexpressing mice showed significant deficit of learning than did the control mice (*p* = 0.0015; Fig. 2B). In the memory trials of the MWM test, Sema3D-overexpressing mice were inferior to the control group in both latency (*p* = 0.0025; Fig. 2C) and frequency (*p* < 0.0001; Fig. 2D) to the platform quadrant. Consistently, Sema3D-overexpressing mice showed impaired recognition memory as reflected by a lower discrimination index (*p* = 0.0007; Fig. 2E); additionally, Sema3D-overexpressing mice might also exhibit worse working memory (Figure S3F) although the difference was not statistically significant due to a large variation (*p* = 0.071). Given that Sema3D overexpression in the hippocampus did not affect the locomotor activity (*p* = 0.248; Figure S3G), the negative effects of Sema3D on the above cognitive function tests would not be related to mobility.

To further confirm the chronic effect of over-expression of cerebral Sema3D on cognitive decline, bilateral intracerebroventricular (ICV) injection of Lv.Sema3D was performed biweekly for a total of 5 injections from week 0 to week 8. The Y-maze test was carried out on the 4^th^ day after each ICV injection to measure the sequential changes in spatial working memory, with the result of one-way repeated measures ANOVA showing that mice with chronic increase of Sema3D exhibited time-dependent cognitive decline (*p* = 0.014, Fig. 2F) and post-hoc analysis illustrating significant cognitive decline by week 8 (an adjusted *p* value of 0.032) and more deterioration by week 10 (an adjusted *p* value of 0.016). On the other hand, the mice receiving Lv.Ctrl had no change of cognitive function during the 10-week study (*p* = 0.971). Two-way repeated measures ANOVA analysis indicated a significant difference of the change in spatial working memory between these two types of mice (*p* = 0.010, Fig. 2F). When lentivirus was no longer injected to the brain after week 8, the cognitive function of the mice in the Lv.Sema3D group recovered gradually and returned to the baseline after week 18 (Figure S3H). Our findings provide another piece of evidence to support high expression of Sema3D in the brain as a risk factor for cognitive decline.

A rescue study was conducted to further establish the causal relationship between Sema3D and cognitive impairment. We used a single injection of siRNA-Sema3D to the hippocampus of miR-195a KO mice aged 12 months. The efficiency of siRNA-Sema3D was evaluated by immunofluorescence analysis (Figure S4A) while spatial working memory and locomotor function were assessed by the Y-maze test (scheme depicted in Figure S4B). Consistently, knockdown of Sema3D in the hippocampus improved spatial working memory (*p* = 0.042 by two-way repeated measures ANOVA, Fig. 2G and Supplementary Table S1). Furthermore, the therapeutic effect of siRNA-Sema3D was also demonstrated by an increased density of dendritic spine in the hippocampal neurons (Figure S4C). Similar to the effect of Sema3D over-expression, siRNA-Sema3D did not appear to influence locomotor function (Fig. 2H) given that siRNA was deliberately injected into the hippocampus.

Taken together, the results from histological examination and behavioral tests suggest that Sema3D is a cause for cognitive impairment and neurodegeneration; furthermore, suppression of Sema3D possesses potential in treatment for dementia.

### High Sema3D level reduces life span and impairs climbing activity in *Drosophila*

**Fig. 3:**
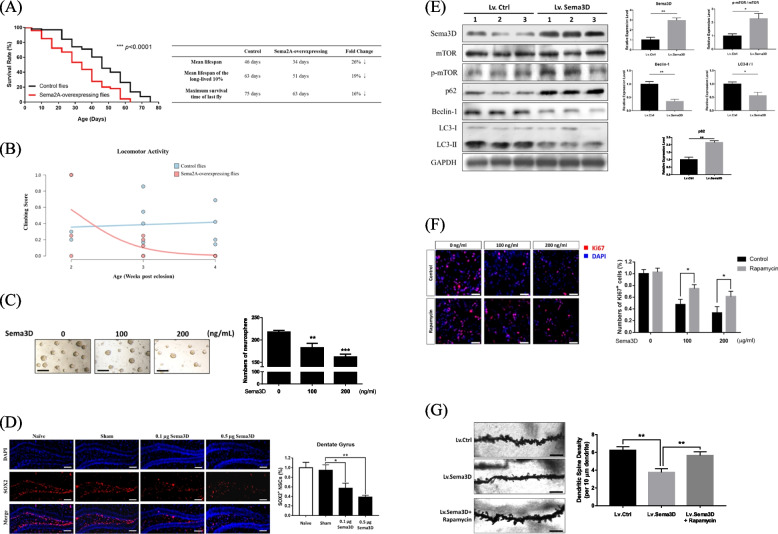
Sema3D affects neural stem cells and autophagy. **A** Survival curve of Sema2A-overexpressing flies and control flies (*n* = 300/ each group) was analyzed by the Gehan-Breslow-Wilcoxon test. The data showed Sema2A-overexpressing flies had shorter life span than the control flies. **B** Adult Sema2A-overexpressing flies show age-related locomotion decline by the negative geotaxis assay at 2, 3, and 4 weeks-post eclosion (*n* = 150/group; *p* = 0.020, Mann–Whitney test). **C** Representative images of neurosphere formation by human NSCs at 48 h after Sema3D treatment. Scale bar = 200 μm. Quantitative data are presented as mean ± SEM from three independent experiments. **p < 0.01 and ***p < 0.001. **D** Sema3D decreased the NSC population in a dose-dependent manner. Representative images of SOX2 + (red) NSCs in the hippocampal dentate gyrus (DG) of mice receiving Sema3D injection (left panel). Magnification: 20X. (Scale bar = 200 μm; *n* = 3/group). Quantification of SOX2 + cells is shown on the right (mean ± SEM) from three independent experiments. *p < 0.05 and **p < 0.01. **E** Lv.Sema3D or Lv.Ctrl was bilaterally injected into the hippocampus of 4-month-old WT mice. On Day14, Sema3D and autophagy-associated proteins in the hippocampus were measured by western blot (*n* = 3/group). **F** Rapamycin reversed the inhibitory effect of Sema3D on cell proliferation in human neurons (SY5Y) by simultaneous treatment of Sema3D and rapamycin for 72 h. The proliferation of neurons was determined using Ki67 staining. Representative fluorescence images of Ki67 and DAPI staining. Scale bar = 50 μm. Quantitative data are presented as mean ± SEM from three independent experiments. *p < 0.05 and **p < 0.01. **G** Lv.Sema3D with/without rapamycin (0.2 nmol/0.2 µL) or Lv.Ctrl was bilaterally injected into the hippocampus of 4-month-old WT mice. The dendritic spine density was measured on Day 14. Representative images of apical dendritic shaft of CA1 pyramidal neurons (*n* = 3 mice/group; 15 neurons/mice). Scale bar = 5 μm. **p < 0.01

Mounting evidence supports the notion that neurodegeneration can reduce the lifespan [31–33]. We used a *Drosophila* model to test the association between Sema3D expression and lifespan, and since the *Sema2A* gene in *Drosophila* is the homology of the *Sema3D* gene in humans and mice, we overexpressed Sema2A in the nervous system of *Drosophila* (denoted as Sema2A-overexpressing flies). As shown in Fig. 3A, Sema2A-overexpressing flies had a significantly shorter lifespan than control flies (*n* = 300/each group, *p* < 0.0001). The mean lifespan of the Sema2A group was decreased by 26% and the maximum lifespan was also significantly diminished from 75 to 63 days. This decrease of life span in *Drosophila* is consistent with the life span data on miR-195a KO mice (Figure S1E). In addition, we used the climbing assay in adult flies to assess whether the neural functions were intact because locomotion as a behavioral output requires fine coordination of neural modalities and is sensitive to functional disruption. The Sema2A-overexpressing flies had reduced locomotor function as shown by reduction in climbing scores (*p* = 0.020; Fig. 3B).

### Mechanisms of the detrimental effects of Sema3D on the brain

We explored two possible mechanisms for the detrimental effect of Sema3D, which are function of NSC and autophagy.

#### Neuro-regeneration

The function of NSC is related to neuro-regeneration. We speculated whether Sema3D could disrupt NSC functions to account for its effect on neurodegeneration and also to partially explain a low NSC population density in miR-195a KO mice (Fig. 1F and Figure S1H). A neurosphere formation assay was conducted and the number of NSCs was quantified with results showing that Sema3D dose-dependently inhibited neurosphere formation by human NSCs, suggesting Sema3D impaired stemness of NSCs (Fig. 3C). When recombinant Sema3D was bilaterally ICV-injected into the mice, the NSC population size in the DG and SVZ was dose-dependently reduced (DG and SVZ results in Figs. 3D and S4D respectively); accordingly, Sema3D reduced NSC stemness and caused NSC loss, which might contribute to neurodegeneration.

#### Autophagy

Disruption of autophagy plays an important role in neurodegenerative disorders [34] and the PI3K/AKT/mTOR pathway is reported as a major regulator in the autophagic process [35], so we hypothesized that Sema3D might induce neurodegeneration by regulating autophagy and the PI3K/AKT/mTOR pathway. To test this, the effect of Sema3D on autophagy efficiency was first explored by overexpressing Sema3D in the hippocampus of mice and treating the SY5Y human neurons with Sema3D. Compared with Lv.Ctrl-treated mice, the Lv.Sema3D-treated mice had higher levels of p-mTOR/mTOR and p62 and lower levels of Beclin-1 and LC3-II/I ratios in the hippocampus (Fig. 3E). Consistently, Sema3D dose-dependently increased p62, and decreased Beclin-1 and LC3-II/I ratios in the Sema3D-treated neurons (Figure S4E). Therefore, both in vitro and in vivo models implied that Sema3D might disrupt autophagy.

Next, we assessed whether the PI3K/AKT/mTOR pathway could be regulated by Sema3D and whether Sema3D-induced neurodegeneration could be rescued by rapamycin, being an mTOR inhibitor, with the results indeed showing that Sema3D dose-dependently increased phosphorylation of PI3K, Akt and mTOR in SY5Y neurons (Figure S4F). Moreover, Sema3D inhibited proliferation of neurons, which was reversed by rapamycin (Fig. 3F). To demonstrate our findings in vivo, a single dose of rapamycin was injected to bilateral hippocampi at 5 min after the bilateral injection of Lv.Sema3D to the same area. Histological images showed that rapamycin reversed Sema3D-induced neural damage as demonstrated by an increased density of dendritic spine structure (Fig. 3G). Taken together, these data indicated that Sema3D impaired autophagy in neurons via the mTOR-dependent pathway, which was then rescued by rapamycin.

### Sema3D is a druggable target for neurodegeneration

We attempted to translate our findings to new drug development, with novel small molecules being developed and tested as to whether Sema3D could serve as a druggable target.

#### Protein homology modeling for Sema3D structure

We first explored Sema3D structure in order to identify the docking site for potential inhibitors. As none of the homo sapiens Sema3 family members have known protein structures, the X-ray crystal structure of Mus musculus Sema3A (PDB code: 4GZ8) was used as the template to predict the Sema3D structure in humans by homology modeling (19). The sequence identity between Mus musculus Sema3A and Homo sapiens Sema3D is 60.04% (Figure S5A). The quality of the predicted Sema3D structure has good integrity according to the Ramachandran plot indicating 83.2% of residues in the most favorable region and 15.5% of residues in the allowed region (Figure S5B).

#### Virtual screening to identify potential inhibitors

**Fig. 4:**
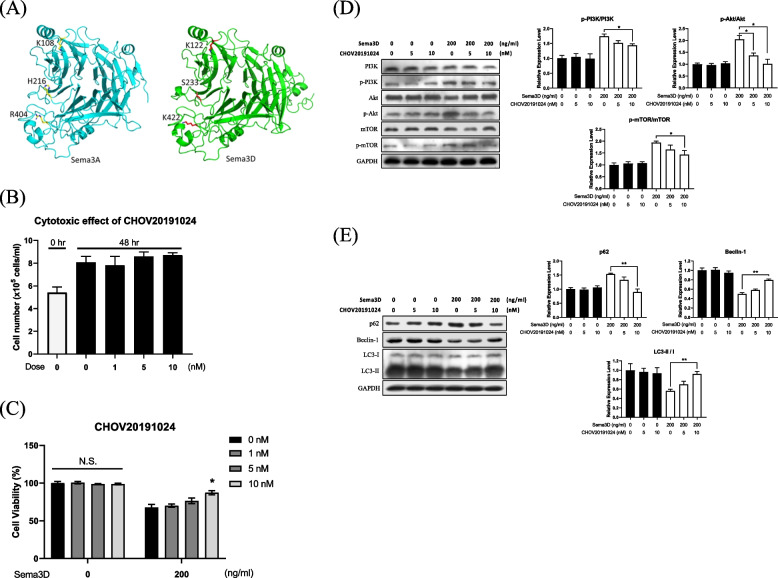
Novel Sema3D antagonist CHOV20191024. **A** Predicted binding site of Sema3D according to the critical residues of Sema3A and PlexinA2 interface. K108, H216, and R404 were on Sema3A; K122, S233, and K422 were on Sema3D. **B** Cytotoxicity of CHOV20191024 was assessed in human neurons (SY5Y) and the total cell number was calculated at 48 h-post-treatment. Quantitative data are presented as mean ± SEM from three independent experiments. **C** CHOV20191024 rescued cell viability of Sema3D-treated SY5Y cells that were simultaneously treated with Sema3D and CHOV20191024 for 48 h. The MTT assay was conducted to determine cell viability. Quantitative western blot data are on the right (mean ± SEM) from three independent experiments. N.S. no significance; *p < 0.05. **D** CHOV20191024 reversed Sema3D-induced signaling cascade in SY5Y cells treated with Sema3D and CHOV20191024 for 24 h. Phosphorylation of the PI3K/Akt/mTOR signaling pathway was measured by western blot. **E** CHOV20191024 rescued Sema3D-induced autophagy dysfunction in SY5Y cells treated with Sema3D and CHOV20191024 for 72 h. Autophagy-associated proteins including mTOR, p62, Beclin-1, and LC3-II / I were measured by western blot. For Fig. 4D and E, quantitative data are presented as mean ± SEM from three independent experiments. *p < 0.05 and **p < 0.01

Xanthofulvin is a Sema3A inhibitor by blocking the binding between Sema3A and Plexin A2 receptor [23]. Three residues (K108, H216 and R404) of Sema3A are critical for the interaction between Sema3A and PlexinA2 receptor [19]. Since the Plexin family is an important receptor for Sema3A, we searched for the critical residues of Sema3D that interact with Plexin receptors, with the results of sequence and structure alignment revealing that K112, S233 and K422 in Sema3D were located in the predicted binding site (Fig. 4A).

Accordingly, we performed virtual screening analysis to identify potential inhibitors to block the binding of Sema3D to the Plexin receptors; additionally, as these inhibitors are unlikely to interfere with other Sema3 receptors such as Nrp1 and VEGFR2 based on the protein sequence alignment, and according to docking simulations, the five most promising inhibitors were identified on the basis of empirical binding energy (kcal/mole). Among these potential inhibitors, compound CHOV20191024 and Sema3D complex was stabilized by three polar contacts. CHOV20191024 interacted with residues I232, S233, and K422 of Sema3D protein, among which S233 and K422 were the critical residues in our predicted binding site. CHOV20191024 exihibited the binding energy of -109.74 kcal/mole with Sema3D.

#### Effect of Sema3D inhibitor

The bioactivity and inhibitory efficiency of CHOV20191024 on Sema3D were explored. Firstly, we demonstrated that CHOV20191024 did not exhibit cytotoxic effect on SY5Y human neurons (Fig. 4B); secondly, we tested whether CHOV20191024 could rescue Sema3D-induced neuron damage with results revealing that CHOV20191024 dose-dependently restored cell viability of Sema3D-treated neurons (Fig. 4C); and thirdly, we explored whether CHOV20191024 could block the Sema3D-induced PI3K/AKT/mTOR activity (Figure S4E) with the data demonstrating inhibitory effect of this pathway (Fig. 4D). CHOV20191024 also rescued Sema3D-induced autophagy dysfunction as evidenced by increased Beclin-1 expression and LC3-II/I ratio (Fig. 4E).

## Discussion

We previously reported that miR-195 has both neuro- and vasculo-protective effects on acute stroke and vascular injury [10, 36]. Moreover, we also reported that miR-195 inhibits age-related blood–brain barrier leakage [26], which further supported the beneficial role of miR-195 in the brain. In the present study, we provide novel findings to suggest that a low level of miR-195 may lead to age-related neurodegeneration and cognitive impairment. To investigate the mechanism accounting for the effect of miR-195 on cognition, we first identified Sema3D as a direct target of miR-195 by conducting a series of experiments to demonstrate the detrimental effects of over-expressed Sema3D on the brain. Initially, we showed that miR-195 levels decreased with age [26] while Sema3D expression increased with age (Fig. 2A and Table 1). Knockdown of cerebral Sema3D rescued spatial memory in miR-195a KO mice, while overexpression of cerebral Sema3D in mice caused significant memory and learning deficits regardless of short-term or long-term elevation of cerebral Sema3D. Both miR-195a KO mice and *Drosophila* with overexpression of Sema3D homology had shorter lifespan and reduced locomotor activity, while over-expression of Sema3D in rodent brain did not reduce locomotor activity. The negative impact of Sema3D on the brain was further demonstrated in human brain samples. According to the GEO datasets, we found that *Sema3D* mRNA levels were significantly increased with the severity of dementia in the hippocampus, prefrontal cortex, visual cortex and cerebellum. Mechanistically, we found that increased expression of Sema3D may impair autophagy and the mTOR pathway. To further support this finding, we used rapamycin, an mTOR inhibitor, to improve autophagy in Sema3D-overexpressing mice. Other mechanisms to account for the detrimental effect of Sema3D included loss of NSCs, impairment of NSC stemness, and reduced density of dendritic spine. To test whether Sema3D is a druggable target, we developed several inhibitors to block Sema3D-induced PI3K/AKT/mTOR signaling, and these inhibitors also restored cell viability of Sema3D-treated neurons. All the aforementioned results offered robust evidence to indicate Sema3D as a novel risk and drug target for dementia and neurodegenerative diseases.

Among Sema3 family members, Sema3A has been reported to play a role in neurodegenerative diseases [15]. Our group recently reported that miR-195 directly targeted Sema3A and mitigated Sema3A-induced neuronal damage [10]. In the present study, we first identified Sema3D as another target of miR-195, and then predicted a significant structural similarity between Sema3A and Sema3D. With additional experiments, we demonstrated that the expression of Sema3D was higher than Sema3A in the aging hippocampus, and Sema3D levels were significantly increased in degenerative brains; therefore, Sema3D could be another undisclosed risk factor for neurodegeneration. Secreted semaphorins could be drug targets for neurodegenerative diseases; for instance, Sema4D antibody was used to rescue cognitive dysfunctions in a mouse model of Huntington disease [37], and depletion of Sema7A has been suggested as a possible treatment for multiple sclerosis [38]. The present study revealed Sema3D is a potential target for cognitive impairment. We designed small molecules to interfere with the binding between Sema3D and its receptor PlexinA without interfering with other Sema3 receptors such as Nrp1 and VEGFR2. Although the clinical applications of our small molecule need to be further tested, the present study sheds light on a new methodology for prevention and treatment of dementia.

Given that the human samples in the GEO databank were likely from chronic patients, it is reasonable that Sema3D was elevated in peri-hippocampal brain regions as the disease progressed. Previous studies have suggested that disruption of the prefrontal cortex and cerebellum directly led to impairment of long-term memory and spatial memory respectively [6, 39], which is compatible with our findings. Moreover, studies have disclosed that disconnection of the hippocampal–prefrontal cortex (PFC) circuit directly impaired cognitive functioning and ability of memory consolidation [40–42]. Determination of whether Sema3D could disrupt the connection between the hippocampus and surrounding cortex is warranted for further investigation.

We are currently optimizing the structure of small molecules to increase the efficacy and improve blood–brain barrier (BBB) penetration. Molecular docking will be experimentally evaluated using the isothermal titration calorimetry (ITC) in the future when the candidate molecule is determined. As such, the current data on small molecules interfering with Sema3D signaling has only demonstrated that continuous endeavor in discovering Sema3D inhibitors for dementia treatment is worthwhile, although we did not explore Sema3D co-receptors because this was beyond the scope of the present work.

Previous knowledge of Sema3D was largely limited to the developing nervous system [43, 44]^.^ Sema3D has been known as a repulsive cue toward specific neuronal populations, but its role in neurodegeneration in the CNS has never been investigated. The present study revealed alterations in autophagy-associated proteins in response to Sema3D treatment, and the administration of rapamycin alleviated Sema3D-induced dendritic spine loss and disruption of cell viability. These results implied the involvement of the mTOR pathway and autophagy dysregulation in Sema3D-induced neurodegeneration. However, limited in vivo evidence highlights the need for further investigation into the underlying molecular mechanisms.

Recently, a study showed that Sema3D and PlexinA1 levels were significantly reduced when zymosan and cAMP analog were used to promote regeneration of crushed optic nerve in a rodent model [45]. Their result is in concert with our finding of loss of dendritic spine by Sema3D over-expression. Taken together, activation of Sema3D/PlexinA1 signaling in the neural system could reduce neural regeneration, thereby facilitating neurodegeneration and eventually causing cognitive dysfunction.

## Conclusion

In conclusion, we initially demonstrated the protective role and anti-aging effect of miR-195 in the brain. Afterward, we identified Sema3D as one of the miR-195-target genes, and determined that Sema3D might cause neurodegeneration via the mTOR/autophagy pathway, impairment of neural stem cells and loss of dendritic spine. Analyzing human brain data also supported that a high level of Sema3D was associated with dementia. This novel finding urged us to develop Sema3D antagonists to block its signaling pathway. Our study provides a pathway in developing new strategies to prevent and/or treat age-related dementia and cognitive impairment.

## Supplementary Information

**Additional file 1.**

## Data Availability

The datasets supporting the conclusions of this article are included within the article and its additional files.

## Abbreviations

Sema3D: Semaphorin3D
AD: Alzheimer’s disease
FTLD: Frontotemporal lobar degeneration
NSC: Neural stem cell
WT: Wild-type
KO: Knockout
MWM: Morris Water Maze
OFT: Open field test
DG: Dentate gyrus
SVZ: Subventricular zone
SA-β-gal: Senescence-associated β-galactosidase
ICV: Intracerebroventricular
