# Supplementary material for: Cerebral Semaphorin3D is a novel risk factor for age-associated cognitive impairment
Source: Cell Commun Signal. 2023 Jun 14;21:140. doi: 10.1186/s12964-023-01158-5 (PMC10265764; doi:10.1186/s12964-023-01158-5)
Supplement: Supplementary file 2 — Additional file 1. [file 12964_2023_1158_MOESM1_ESM.docx]

**Supplemental Figures and Tables**

**Materials and Methods**

**Fig S1.** Characteristics of miR-195a KO mice

**Fig S2.** miR-195 and Sema3D

**Fig S3.** The effect of Sema3D on the brain

**Fig S4.** The effect of Sema3D on neural cells and autophagy-associated PI3K/Akt/mTOR pathway

**Fig S5.** Prediction of Sema3D structure.

**Table S1.** Results of behavioral tests in miR-195a KO mice, Sema3D-overexpressing mice, and Sema3D siRNA-injected mice.

**Table S2.** Available GEO datasets on gene expression in hippocampus, cortex, and cerebellum.

**Table S3.** Stereotactic coordinates for all experiments in the present study.

**Materials and Methods**

**Reagents**

MiR-195 and negative control microRNA (NC-miR) were purchased from Ambion Inc. (Austin, TX, USA) with the sequence’s information: miR-195 mimic, 5’-UAGCAGCACAGAAAUAUUGGC-3’; anti-miR-195, 5’-GCCAATATTTCTGTGCTGCTA-3’; negative control sequence, 5’-AGUACUGCUUACGAUACGG-3’. SYBR^®^ Green PCR Master Mix, MultiScribe^®^ Reverse Transcriptase Kit were purchased from Applied Biosystems (Foster City, CA, USA). Rapamycin, a chemical mTOR inhibitor, was purchased from Sigma Aldrich (St. Louis, MO, USA). Recombinant mouse Sema3D and human Sema3D proteins were purchased from R&D Systems (9386-S3-025 and 9674-S3-025; Minneapolis, MN, USA). Unless otherwise specified, all other reagents were of analytical grade. Primary antibodies anti-SOX2 (ab97959, Abcam; Cambridge, MA, USA), anti-Sema3D (ab180174 for western blot and ab198849 for immunofluorescence stain, Abcam), anti-Sema3A (ab199475, Abcam), anti-GAPDH (5174, Cell Signaling; Beverly, MA, USA), anti-Ki67 (ab16667, Abcam), anti-Beclin-1 (ab207612, Abcam), anti-LC3 (ab48394, Abcam), anti-p62/SQSTM1 (ab56416, Abcam), anti-PI3K (E-AB-32575, Elabscience; Houston, Texas, USA), anti-phospho-PI3K (E-AB-20966, Elabscience), anti-Akt (9272S, Cell Signaling), anti-phospho-Akt (9271S, Cell Signaling), anti-mTOR (E-AB-32128, Elabscience) and anti-phospho-mTOR (E-AB-20929, Elabscience) were used in western blot, immunohistochemical staining, and immunofluorescence experiments.

**Generation of miR-195a KO mice**

MiR-195a KO mice on C57BL/6 background were generated by the National Laboratory Animal Center, Taiwan. To generate miR-195a knockout mice, a mouse bacterial artificial chromosome (BAC) clone RP23-301F19 covering the whole miR-195a gene was obtained from BACPAC Resources Center at the Children’s Hospital of Oakland Research Institute. A loxP and a frt-neo-frt-loxP selected marker were inserted upstream and downstream of the miR-195 locus in the BAC clone using the RED/ET recombination technique (Gene Bridges, Heidelberg, Germany) so as to generate the targeting construct. The detailed protocol is described in the instruction manual of the counter selection BAC modification kit (Gene Bridges, Heidelberg, Germany). The modified BAC construct was purified, *Not* I digest, and phenol/chloroform extraction used to isolate the linearized DNA for electroporation of C57BL/6 mouse embryonic stem cells. Embryonic stem (ES) cell electroporation, selection, and screening were performed using standard gene targeting techniques. All of the PCR products amplified from positive clones for homologous recombination were confirmed by sequencing. Positive clones were injected into C57BL/6 albino (B6(Cg)-Tyrc-2J) blastocysts to generate chimera, which were crossed with C57BL/6 albino for germ-line transmission breeding test.

**Sema3D-overexpressing mice and lentivirus administration**

4-month-old C57BL/6 mice were used to generate Sema3D-overexpressing mice. Stereotaxic injection procedures were performed on anesthetized mice using a mixture of Zoletil and Rompun (3:1 ratio, 1 mg kg^−1^, intraperitoneally). A solution containing Sema3D lentivirus (Lv.Sema3D; 4.5 × 10^5^ TU/ml) was prepared to over-express Sema3D in the brain.

For mice receiving injection to the bilateral hippocampi, we injected the lentivirus to the anterior and posterior of each hippocampus. (−1.2 mm anterior–posterior, 1 mm medial–lateral, and −2 mm dorsal–ventral relative to the bregma; −3.6 mm anterior–posterior, 3.2 mm medial–lateral, and −4 mm dorsal–ventral relative to the bregma). On Day7 post-injection, these mice were subjected to behavioral tests, Golgi-cox stain, or signal pathway analysis. The mice receiving control lentivirus (Lv.Ctrl) from hippocampal injection served as the control group.

For the study of chronic over-expression of Sema3D, two small holes were drilled (-0.9 mm posterior to bregma, ±1.7 mm lateral to the sagittal suture; 2.2 mm in depth) in the skull, and a solution containing Sema3D lentivirus was bilaterally intracerebroventricularly (ICV) injected every two weeks. The Y-maze test was carried out on Day 4 after each ICV injection for a total of 10 weeks to measure the sequential changes in spatial working memory. The mice receiving control lentivirus (Lv.Ctrl) from intracerebroventricular injection served as the control group.

The materials to produce lentivirus were commercially available and the lentivirus was made in our lab. Mouse Sema3D cDNA ORF clone was purchased from Origene (NM_028882) and constructed into a lentivirus-expressing vector using a lentivirus-expressing system (cat. no. K532000; Invitrogen; Thermo Fisher Scientific, Inc.) according to the manufacturer's protocols.

**Hippocampal injection of siRNA**

Sema3D siRNA or control siRNA was delivered into the hippocampus of 12-month-old miR-195a KO mice. Stereotaxic injection procedures were performed on anesthetized mice by using a mixture of Zoletil and Rompun (3:1 ratio, 1 mg kg^−1^, intraperitoneally). A solution containing Sema3D siRNA or Control siRNA (0.2 nmol/injection site) was injected to bilateral hippocampi. To knockdown Sema3D in the entire hippocampus, we injected the lentivirus to the anterior and posterior of each hippocampus. (−1.2 mm anterior–posterior, 1 mm medial–lateral, and −2 mm dorsal–ventral relative to the bregma; −3.6 mm anterior–posterior, 3.2 mm medial–lateral, and −4 mm dorsal–ventral relative to the bregma). Mice were subjected to behavioral tests from Day 7 post-injection, and subjected to Golgi-cox stain on Day 15. The mice receiving control siRNA (si-Ctrl) served as the control group.

**Y-maze**

The spatial working memory was assessed by the Y-maze test. The Y-maze consisted of three enclosed arms, 50 cm long, 11 cm wide and 10 cm high made of black Plexiglas, set at an angle of 120° to each other, in the shape of a Y. Two different Y-maze protocols were used in the present study.

The first protocol, which consisted of two trials separated by a 15-min interval, was carried out in miR-195a KO mice and Sema3D-overexpressing mice. In brief, in the first trial (acquisition trial), mice were placed at the end of a chosen arm (start arm) and allowed to explore the maze for 5 min with one of the arms closed (denoted as novel arm). Mice were then returned to their home cage located away from the test room for 15 minutes. In the second trial (retention trial), the mice were allowed to explore all three arms of the maze freely for 5 min, and the time spent to reach the novel arm (previously closed in the first trial) was recorded. The longer the time recorded to reach the novel arm, the worse the performance of working memory.

The second protocol was carried out in Sema3D/control siRNA-treated miR-195a KO mice. In brief, each animal was placed in the center of the Y-maze and was free to explore the arena for 8 minutes. Mice tended to explore the least recently visited arm, and thus tended to alternate visits between the three arms. For efﬁcient alternation, mice need to use working memory and they should maintain an ongoing record of most recently visited arms while continuously updating such records. An arm entry was scored when mice placed the four paws within that arm. Spatial working memory was assessed by percentage of alternation deﬁned as an entry into three different arms on consecutive choices, then the percentage of alternation was calculated as the ratio of actual to maximum number of alternations corresondingly deﬁned as total number of arm entries minus 2. A low percentage of alternation is indicative of impaired spatial working memory because the mouse would be unable to remember which arm it had just visited, thus showing decreased spontaneous alternation. Locomotor function was determined in this Y maze test based on the total travel distance, with longer distance indicating better locomotor function.

**Open field test (OFT)**

The locomotor function was examined using the open field test (OFT). Briefly, mice were placed in an open field apparatus equipped with 16 photo beams for a single 15-minute session, then the locomotor function was determined by total travel distance of each mouse.

**Morris Water Maze**

Spatial learning and spatial memory were assessed by the MWM test. In brief, mice were trained to find a hidden platform in opaque water for 5 days with four acquisition-trials per day from pseudorandomized start positions. We set 5 minutes to let the mice freely explore the platform in learning trial and memory trial. If the mouse could not find the platform in 5 minutes, the experiment would be stopped to prevent the mouse from drowning. During the 5-day acquisition trials, latency to find the hidden platform was recorded as an index of spatial learning ability. Next, to assess spatial memory ability, probe trials (where the submerged platform was removed) were performed on days 1, 7, and 14 after acquisition trials whereby total time spent to find the hidden platform was recorded. Latency to find the hidden platform and frequency to reach the platform quadrant were recorded as indexes of spatial memory ability. All MWM trials were recorded and analyzed using the ANY-Maze tracking system (Stoelting, Chicago, IL, USA).

**Novel Object Recognition test**

Recognition memory was assessed by the Novel Object Recognition Test. Mice were first placed in the center of the arena with two identical objects for 10 minutes. Mice were then returned to their home cage located away from the test room for another 15 minutes. Next, recognition memory was tested in a 5-minute session for which one of the familiar objects was replaced with a novel object. The time each animal spent exploring each object during testing was recorded with a video tracking system (ViewPoint Behavior Technology; Lyon, France.). Object memory ability was revealed as the proportion of time spent exploring the novel object compared to the time spent exploring all objects (discrimination index).

**Golgi-cox staining and dendritic spine density measurement**

Dendritic spine density of hippocampal neurons in CA1 region was revealed by Golgi-cox stain. Brains were immersed in the Golgi staining solution according to the manufacturer’s protocol (FD Rapid GolgiStainTM Kit, FD NeuroTechnologies Inc., MD, USA); then coronal sections of 100 µm thickness were sliced from the dorsal hippocampus using a vibratome (Leica VT1000S) and mounted on gelatin-coated slides, followed by 15 min in Kodak Film Fixer and dehydrated with a xylene-based medium.

Bright-field images were acquired using confocal microscope (Leica SP2 / SP8X). For dendritic spine density analysis, 3 brain slices (−1.20 ~ −2.50 mm anterior–posterior relative to the bregma) were selected per animal; and 5 neurons per slice were analyzed (15 neurons/animal). Hippocampal CA1 dendrites were semi‑automatically traced with the ImageJ software. Pyramidal neurons that were minimally obscured by other cells and had an unimpaired dendritic tree were then selected for analysis.

**Immunofluorescence stain and quantification of neural stem cells (NSCs)**

To evaluate neurogenesis ability *in vivo*, NSC was detected using immunofluorescence staining. NSCs were confirmed and quantified by SOX2 positive signals with clearly discernible nuclei (DAPI positive cells). Briefly, brains were fixed with 4% PFA, cryopreserved for 24 h at 4°C in 30% sucrose and embedded in OCT. Fifteen-μm-thick cryosections were collected and stored at -20°C until use. For SOX2-positive cell staining and quantification in dentate gyrus (DG) and subventricular zone (SVZ), three brain slices with DG (−1.20 ~ −2.50 mm anterior–posterior relative to the bregma) were selected per animal, and three brain slices with SVZ (+1.00 ~ +0.00 mm anterior–posterior relative to the bregma) were selected per animal for immunofluorescence stain. To perform immunostaining, the brain slices were incubated with SOX2 antibody in PBS with 5% BSA overnight at 4°C and incubated with secondary antibody conjugated by Alexa Fluor 647 (Invitrogen). Images were obtained by an immunofluorescence confocal microscope (Leica SP2 / SP8X). The number of SOX2-positive cells located in the dentate gyrus (DG) and subventricular zone (SVZ) were counted and quantified by ImageJ using 4-month-old WT mice as the reference group. The number of SOX2 positive cells located in the dentate gyrus (DG) and subventricular zone (SVZ) were quantified by the ImageJ software.

**Intracerebroventricular (ICV) injection of recombinant Sema3D protein**

To explore the effect of Sem3D on neurogenesis, recombinant Sema3D protein was dissolved in PBS and subjected to 4-month-old WT mice by ICV injection. To conduct ICV injection protocols, mice were anesthetized by isofluorane (1.2 ± 0.2%) and the head was secured in a stereotaxic apparatus (Stoelting, Wood Dale, IL, USA). Following the incision and exposing the skull, two small holes were drilled (-0.9 mm posterior to bregma, ±1.7 mm lateral to the sagittal suture). A 33-gauge needle was lowered into the ventricles (2.2 mm in depth) and Sema3D protein was infused, the needle was removed slowly to avoid infuscate backflow and the skull was closed with bone wax and sutured. The effect of Sema3D on neurogenesis was assessed on day 5 post-injection by quantifying SOX2 positive NSCs in the DG and SVZ.

**Sphere formation assay**

The sphere formation assay was used to determine the effect of Sema3D on NSC stemness. In brief, human NSCs were seeded on ultra-low attachment 24-well plates (Corning; NY, USA.). The number of spheres (>50 μm in diameter) was counted on day 5 of culturing.

**Gene expression data acquired from Allen Brain Atlas**

To investigate *Sema3A* and *Sema3D* expression levels in the human hippocampus, RNA sequencing data was downloaded from the Allen Brain Atlas (<https://portal.brain-map.org/>). RNA sequencing data among 94 donors with age 77~100+ years were analyzed for Sema3A and Sema3D gene expression in the present study.

**Senescence-associated β-galactosidase (SA β-gal) activity analysis**

Senescence-associated (SA) β-galactosidase activity was determined using SPiDER-βGal assay kit and X-gal based staining. Briefly, brain slices were first fixed in PBS containing 4% paraformaldehyde. After washing with PBS three times, 5 μm brain slices were exposed to either X-gal solution (1 mg/ml X-gal; 5 mM K_3_Fe(CN)_6_; 5 mM K_4_Fe(CN)_6_; 1 mM MgCl_2_, in PBS; pH=6.0) or SPiDER-βGal staining solution. Slides were mounted and signals in the cortex and hippocampus were investigated using a dissection or immunofluorescence confocal microscope (Leica SP2 / SP8X). Signal intensity of SPiDER-βGal positive cells were normalized by DAPI signal and were quantified by the ImageJ software.

**Target site prediction and luciferase reporter assay**

Two algorithms were used to predict miR-195 target genes and binding sites: miRanda (<http://microrna.sanger.ac.uk/targets/v5/>) and TargetScan (<http://targetscan.org>). The luciferase activity was used to test whether Sema3D was a miR-195 target gene. The reporter plasmid containing the predicted Sema3D binding site (5’- UUCAGCAAUUUA**UGCUGCU**A -3’) or mutant binding site (5’- UUCAGCAAUUUA**GUTGUTG**A -3’) was constructed and transfected into HEK293 cells. After 24 hours, miR-195 or negative control microRNA were transfected into HEK-293 cells by HiPerFect Transfection Reagent (Invitrogen) to investigate whether miR-195 could directly bind to the target 3’-untranslated region (3’-UTR) sequence, then the luciferase activities between the cells transfected with normal or mutant plasmids were compared. If Sema3D was a miR-195 target, the luciferase activity should be higher in the cells transfected with mutant plasmid because miR-195 could not exert its knockdown effect.

**RNA isolation and measurement of mRNA Levels**

Total RNA was extracted from cells using Trizol reagent. A quantitative real-time PCR analysis using cDNA from the cells and tissues was performed using the AB7900 real-time PCR system (Applied Biosystems) according to the manufacturer’s instructions. Specific primers for mouse Sema3A, Sema3D, p16^INK4a^, p19^Arf^, and GAPDH were used, and the ratio of each gene was normalized to the internal control (GAPDH) with expression levels quantified by employing the 2^-△△Ct^ relative quantification method.

**Western blot analysis**

For the western blot analysis, cell pellets or brain samples were homogenized and lysed using RIPA buffer supplemented with protease and phosphatase inhibitors (Complete and Phosphostop, Roche). Twenty micrograms of proteins were subjected to SDS‐PAGE, and electrophoresed proteins were transferred to polyvinylidene-difluoride membrane (Millipore). The immunoblots were incubated with primary antibodies overnight at 4°C, then after washing, they were incubated again with horseradish peroxidase‐conjugated secondary antibodies at 4°C for 1h. Immunoblot analysis was performed using ECL Western blotting detection reagents (GE) according to the manufacturer's instructions.


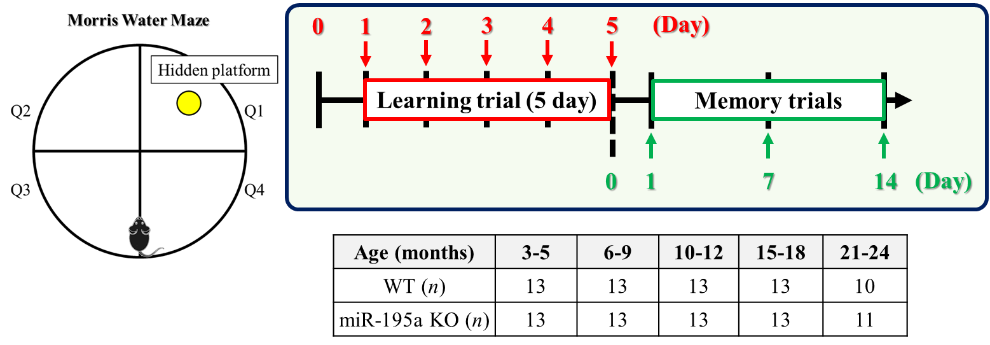
(1A)


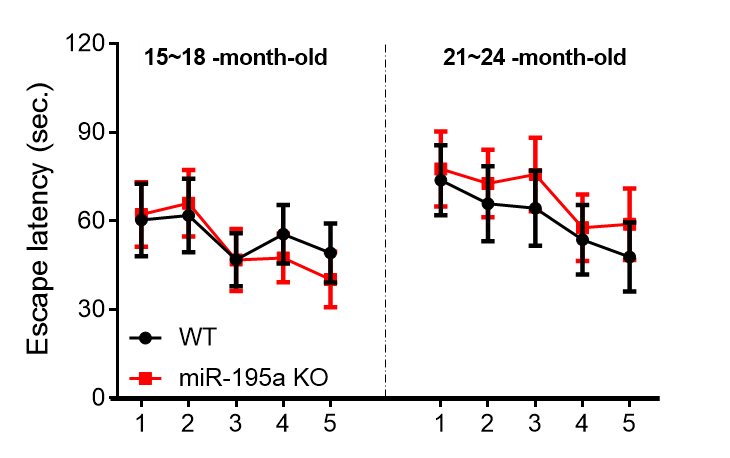
(1B)


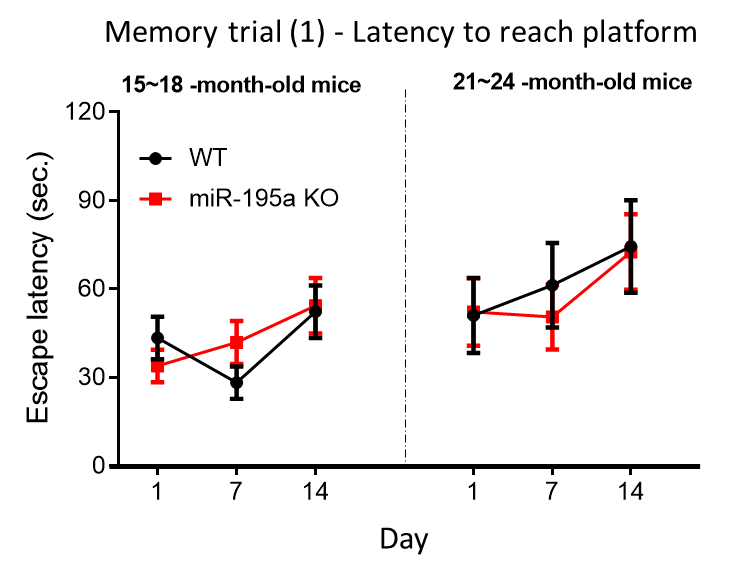
(1C)


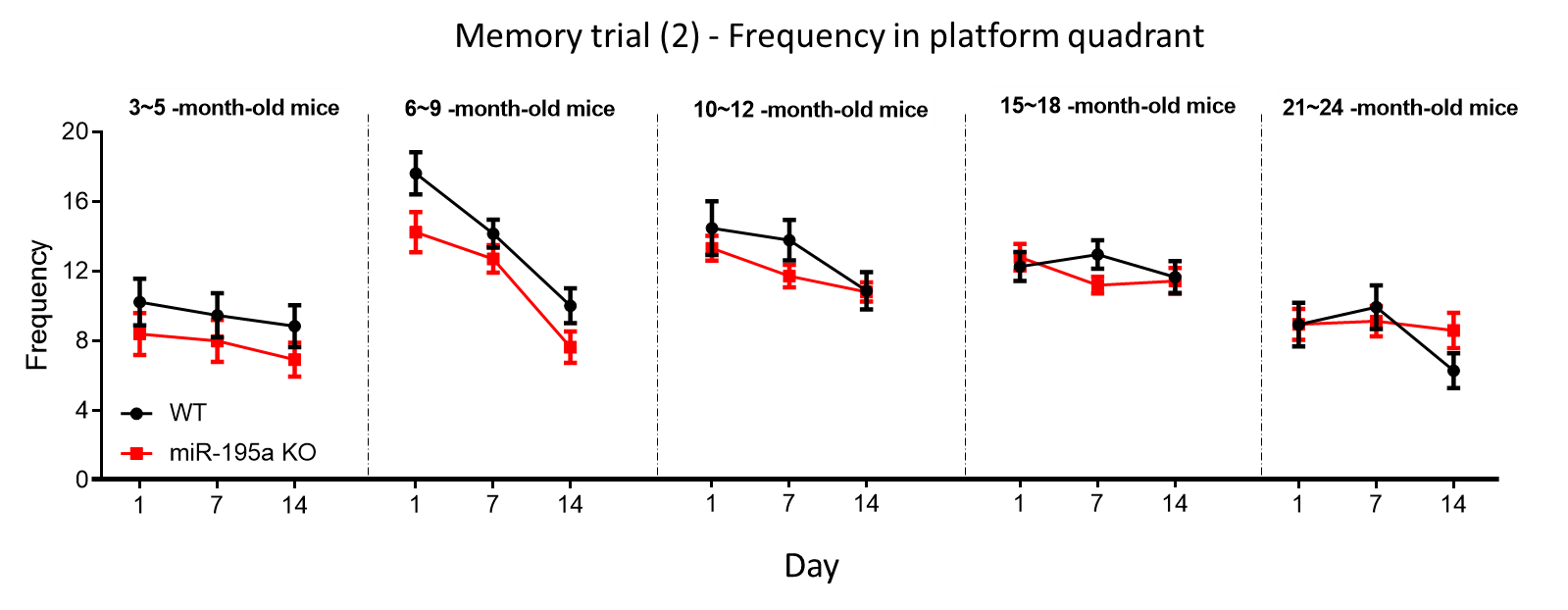
(1D)


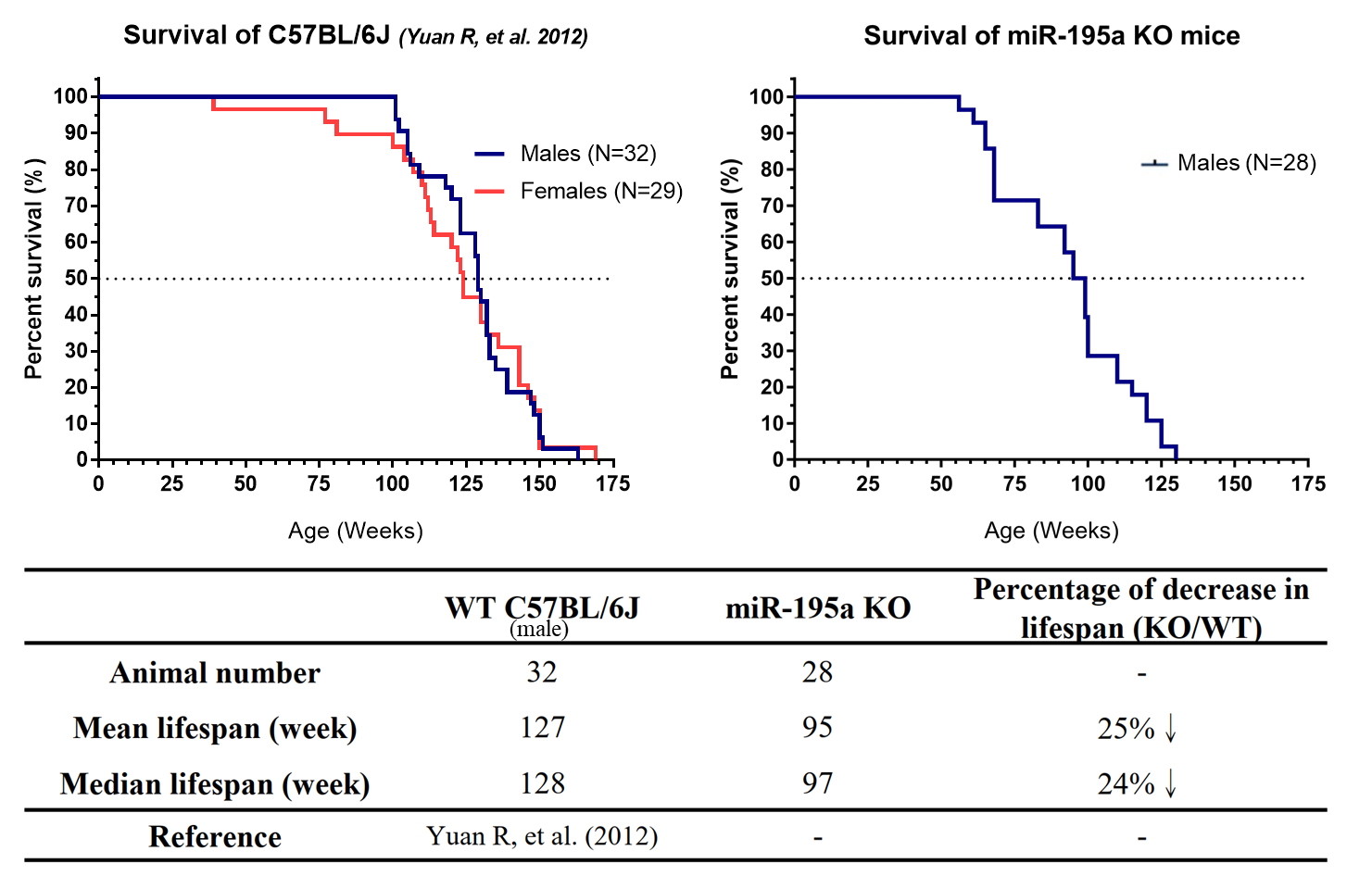
(1E)


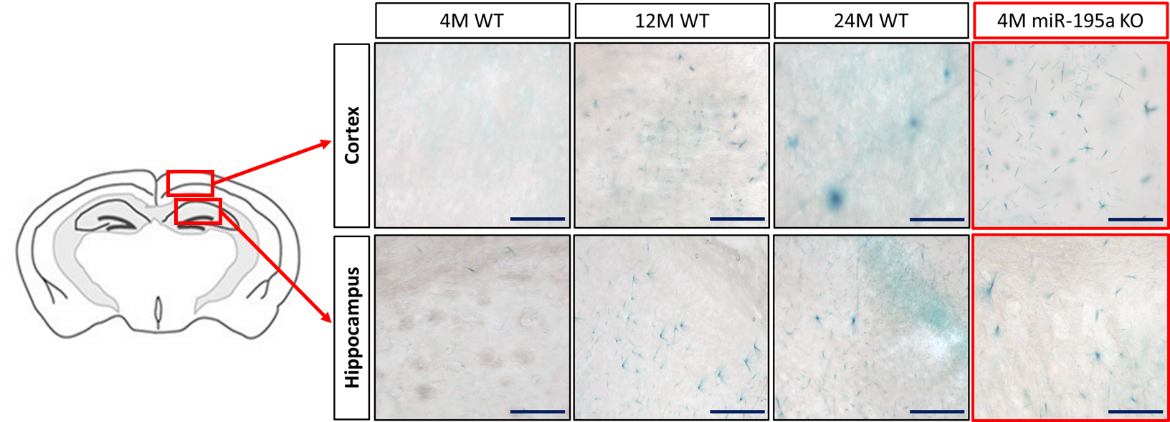
(1F)

(1G)

**
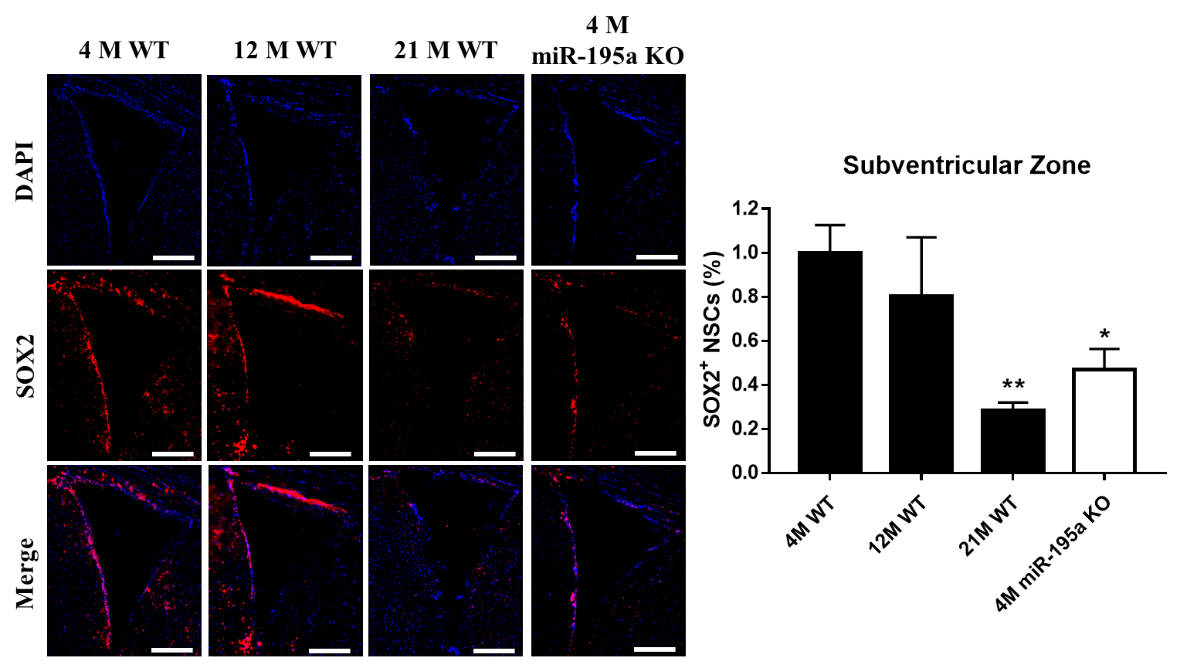
**(1H)

**Supplemental Fig 1. Characteristics of miR-195a KO mice**

(A-C) Learning and spatial memory were evaluated by the Morris Water Maze test (MWM) in miR-195a KO and age-matched WT mice. (A) The scheme for learning trials and memory trials. (B) Learning trial for old miR-195a KO and WT mice. Reaching the hidden platform during training courses. See the data on young and adult mice in Figure 1A. (C) Memory trials for old miR-195a KO and WT mice. The latency for reaching the platform. See the data on young and adult mice in Figure 1C. (D) Memory trials for miR-195a KO and WT mice. The frequency in the platform quadrant. (E) Survival curve of miR-195a KO mice (n=28) and C57BL/6J. The historical data on WT mice reported by *Yuan R. et al.* were used as the reference. Results of mean lifespan, median lifespan and the percentage of decrease are presented in the chart on the lower panel. (F) Senescence associated β-galactosidase (SA β-gal) activity was determined using X-gal based staining. The intensity of green signal represents the activity of SA β-gal enzyme. The representative figures show an age-dependent increase of SA β-gal activity in the cortex and hippocampus of WT. The intensity of green signal is similar between miR-195a KO mice at age of 4 months and WT mice at age of 12 months. Scale bar: 200 μm. (G) Quantitative PCR analysis of p16^Ink4a^ (left panel) and p19^Arf^ (right panel) expression in the whole brain of 4-month-old miR-195a KO mice and age-matched WT mice (n=3/group). Data are presented as mean ± SEM. **p* <0.05; ***p* < 0.01. (H) Representative images of SOX2^+^ (red) NSCs in the subventricular zone (SVZ) of WT mice (the first 3 panels on the left) and miR-195a KO mice (far right images). Quantification of SOX2^+^ cells in the SVZ is shown on the right side. Magnification: 20X. Scale bar = 200 μm. (n=3/group). Data are presented as mean ± SEM. **p* <0.05; ***p* < 0.01 versus 4-month-old WT mice. Data B-H are presented as mean ± SEM from three independent experiments, **p* < 0.05, and ***p* < 0.01.


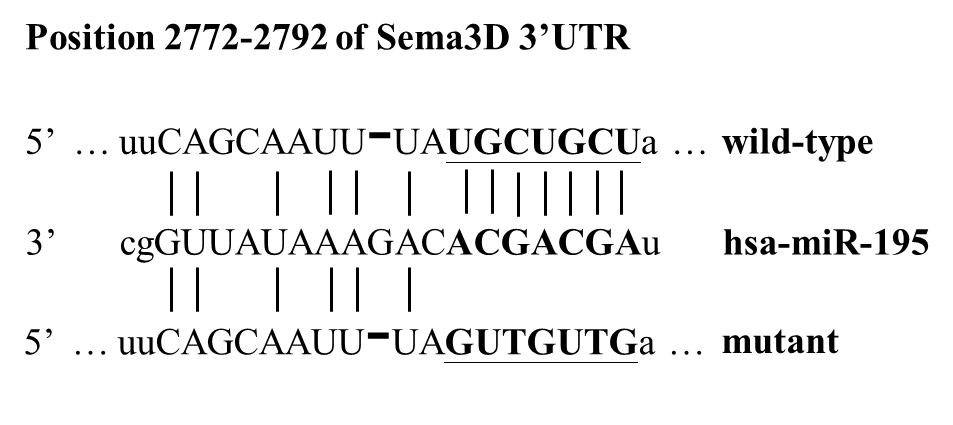

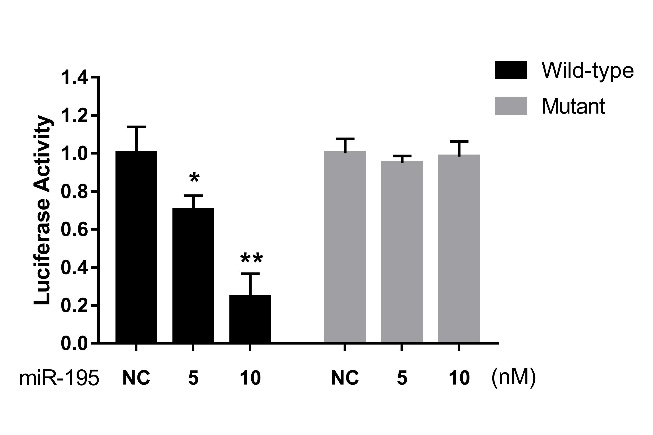
(2A)


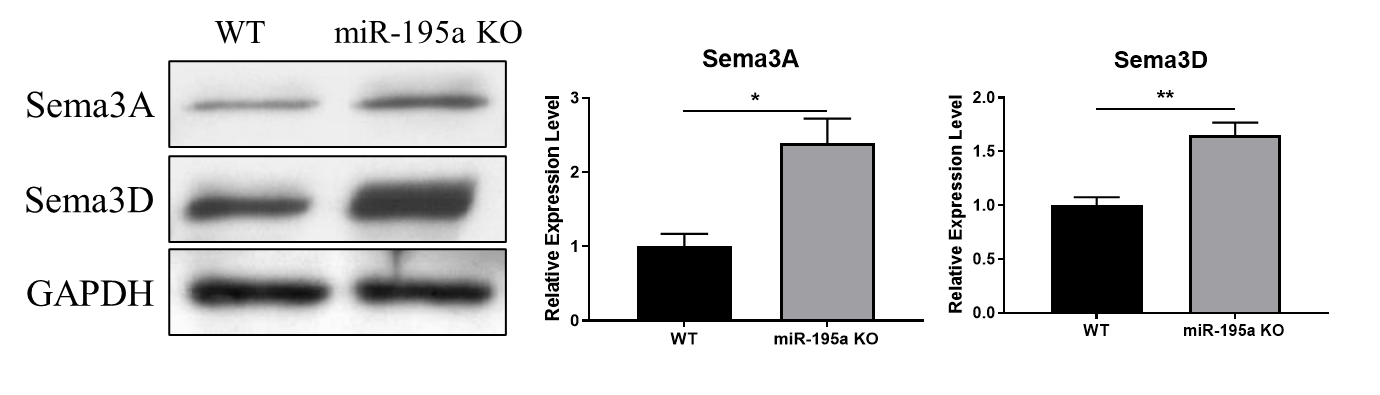
(2B)

(2C)


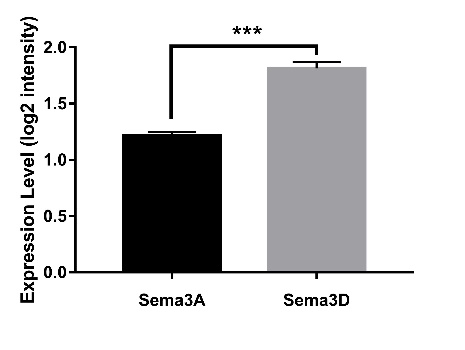
(2D)

**Supplementary Fig 2. miR-195 and Sema3D**

(A) The correspondent seed region in Sema3D 3′-UTR was mutated to disrupt base-pairing between Sema3D and miR-195. The reporter plasmids carrying either wild-type or mutant Sema3D 3′-UTR was transfected into HEK293 cells. miR-195 dose-dependently knocked down the luciferase activity in the cells transfected with plasmids carrying the wild-type Sema3D 3′-UTR (right panel). (B) Western blots and quantitative data of Sema3A and Sema3D protein levels in the hippocampus of WT and miR-195a KO mice aged 4 months (n=3/group). Sema3A and Sema3D in the hippocampus of WT mice were respectively used as the reference groups. (C) Sema3A and Sema3D mRNA expression in the hippocampus of 4-month-old miR-195a KO mice and age-matched WT mice (n=3/group). The WT mice was used as the reference group. (D) Sema3D and Sema3A mRNA expression in human hippocampus (n=95) according to the RNA-seq data from the Allen Brain Atlas. Data are presented as mean ± SEM. *p < 0.05, **p < 0.01. ***p < 0.001


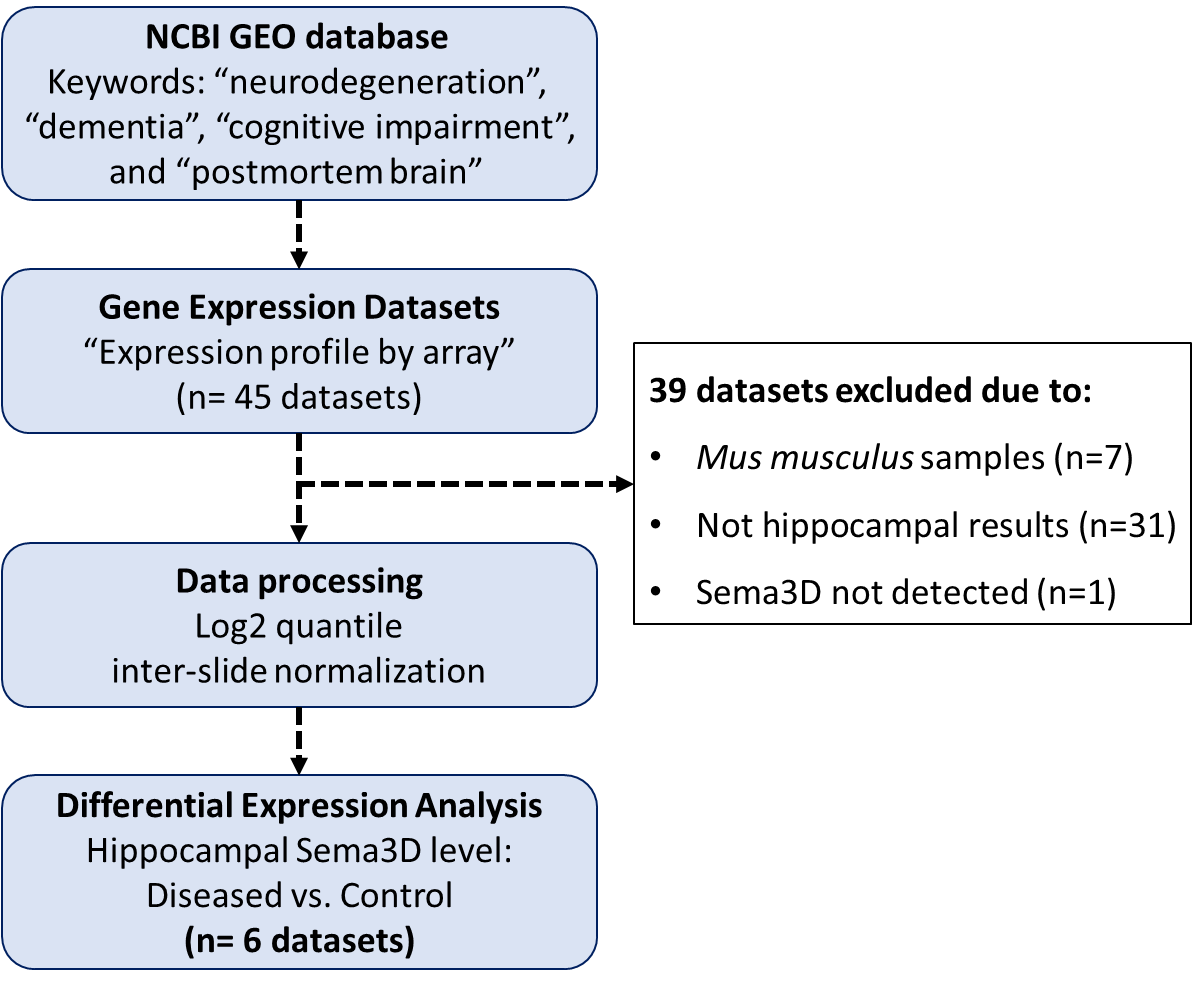
(3A)


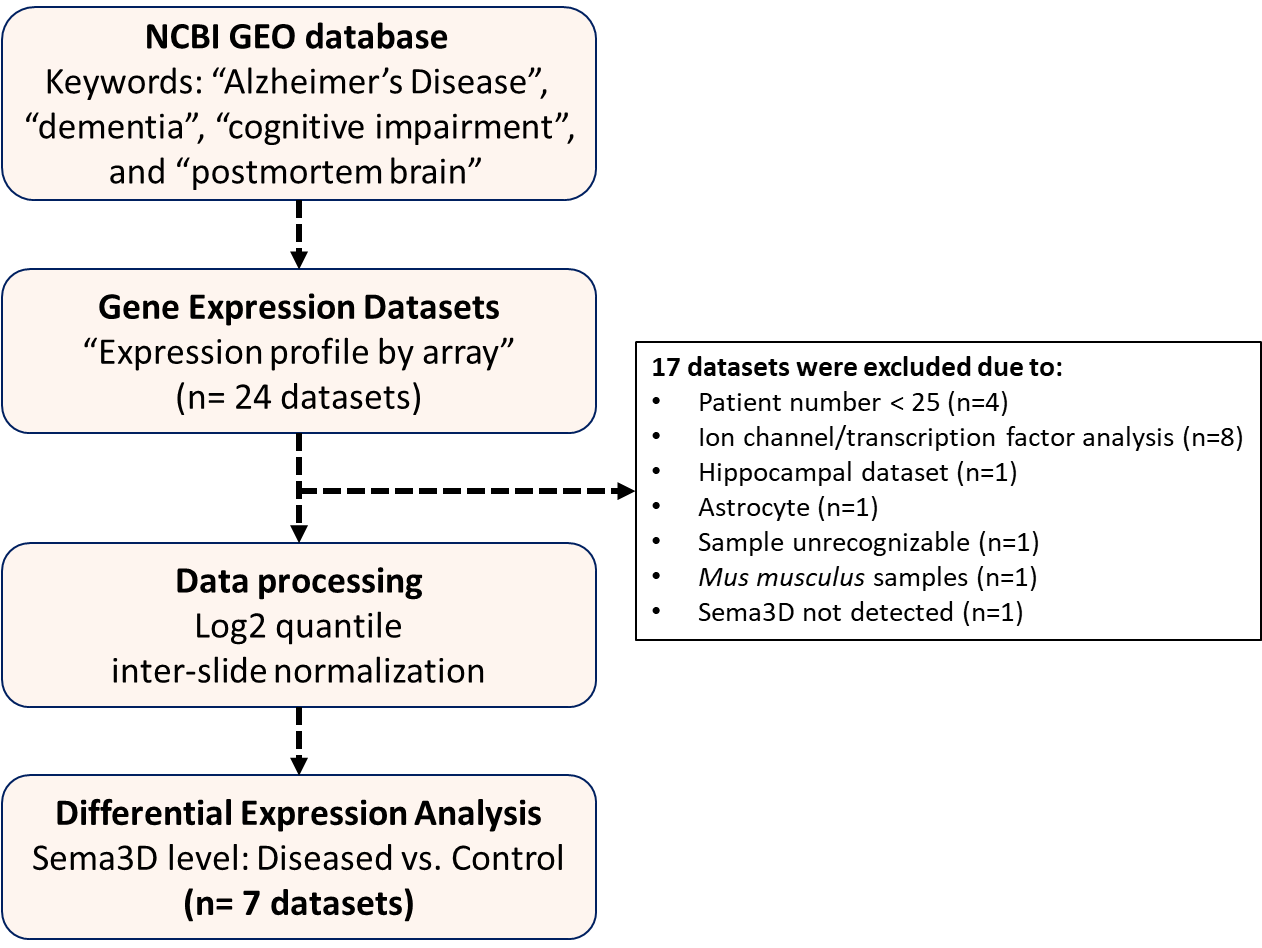
(3B)


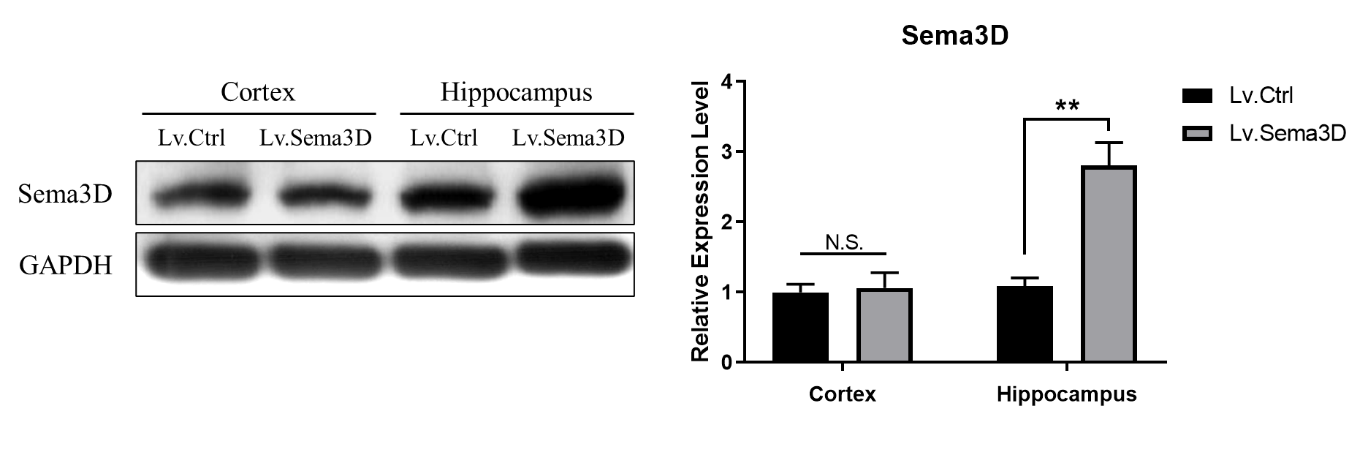
(3C)

(3D)


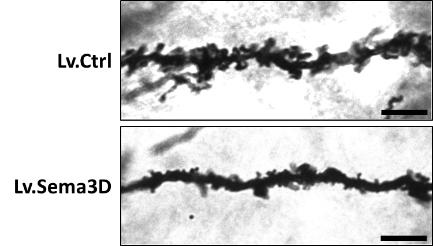


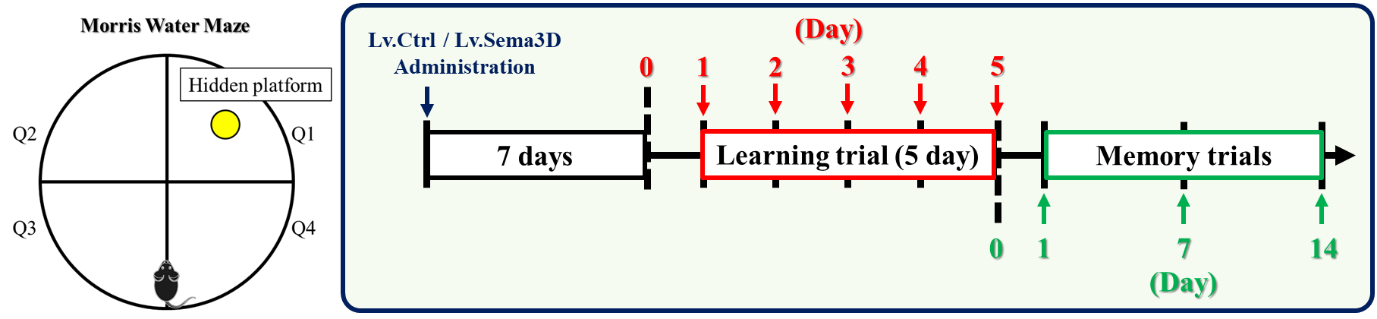
(3E)

(3F)

(3G)

**
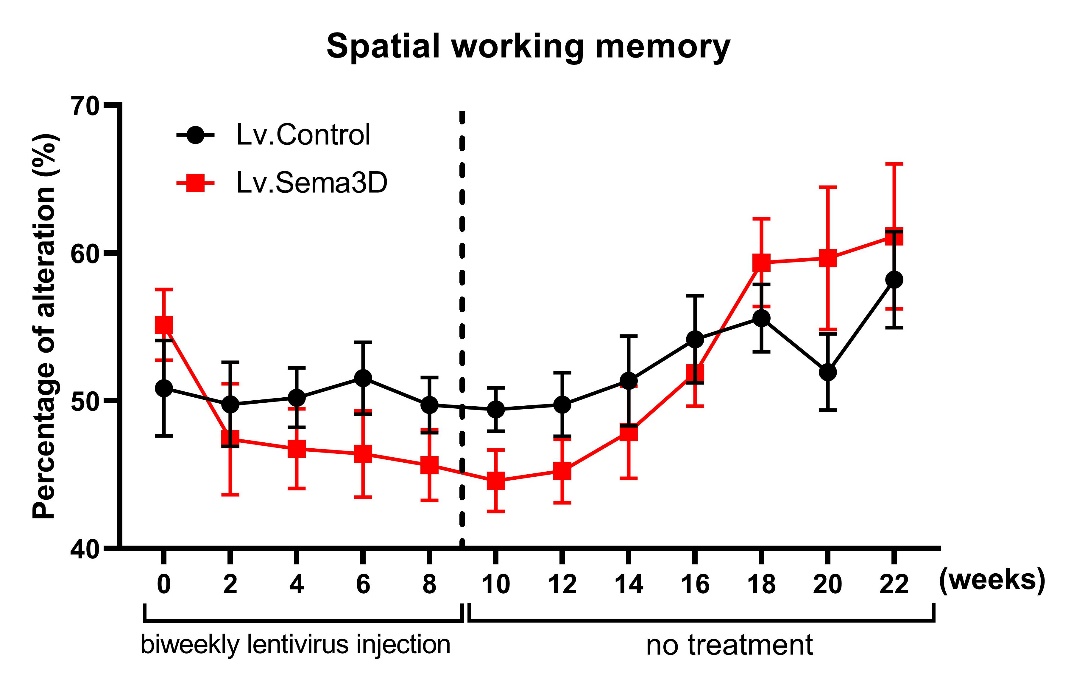
**(3H)

**Supplementary Fig 3. The effect of Sema3D on the brain**

(A-B) Flowchart of the selection and analysis of neurodegenerative diseases transcriptome. The computational analyses consisted of four major steps as presented in the boxes. Detailed criteria for each major step are described in Methods. (C-D) On Day 7, western blotting data showed a higher level of Sema3D protein in the hippocampus but not in the cortex after bilateral injection of Sema3D-expressing lentivirus (Lv.Sema3D) into the hippocampus of 4-month-old WT mice. Injection of control lentivirus (Lv.Ctrl) served as the control group (n=3/group). Quantitative data from western blot are shown on the right. (D) The hippocampus from the same mouse shown in Figure S3B was used for the Golgi-cox stain. The dendritic spine density in the hippocampus was measured using Golgi-cox stain. Representative images show apical dendritic shaft of hippocampal CA1 pyramidal neurons. Scale bar = 5 μm. Quantification of dendritic spine density is shown on the right. (n=3 mice/group; 15 neurons/mice) Data are presented as mean ± SEM. *p < 0.05. (E) Lv.Sema3D or Lv.Ctrl was administered to WT mice; learning trial and memory trials were performed according to the scheme. (F) Memory performance of Lv.Ctrl and Lv.Sema3D-injected mice were examined by Y-maze (n=3/group). Quantitative data showed that Sema3D-overexpressing mice spent more time to reach the novel toy arm, implicating memory impairment (*p* = 0.071). Data are presented as mean ± SEM. (G) Locomotor activity of Lv.Ctrl and Lv.Sema3D-injected mice was measured by the open field test (Lv.Ctrl distance = 116.4±4.01 cm, Lv.Sema3D distance = 100.1±11.35 cm; *p*=0.248; n=3/group). (H) 20 mice (n=10/each group) received 5 lentivirus injections from week 0 to week 8, and the Y maze test was conducted biweekly right before the first injection until week 22. The cognitive function in the Lv.Sema3D group gradually recovered to the baseline after week 18.


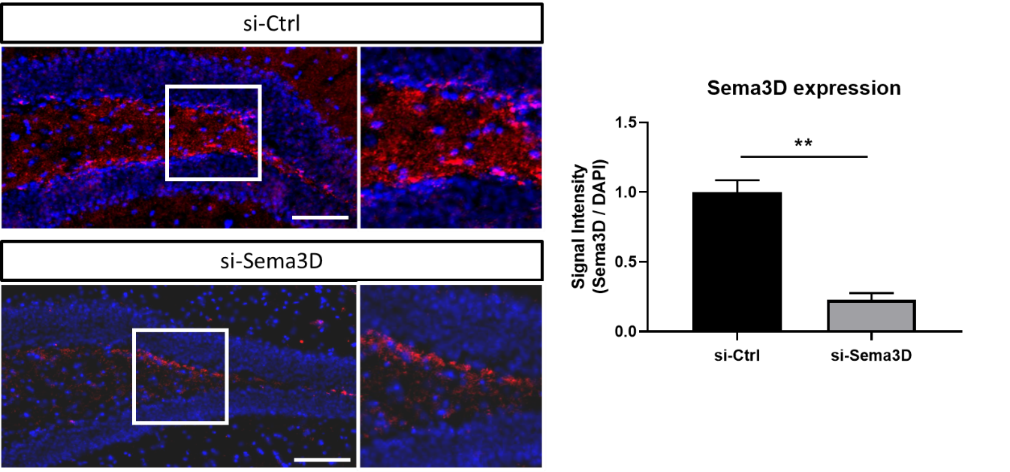
(4A)


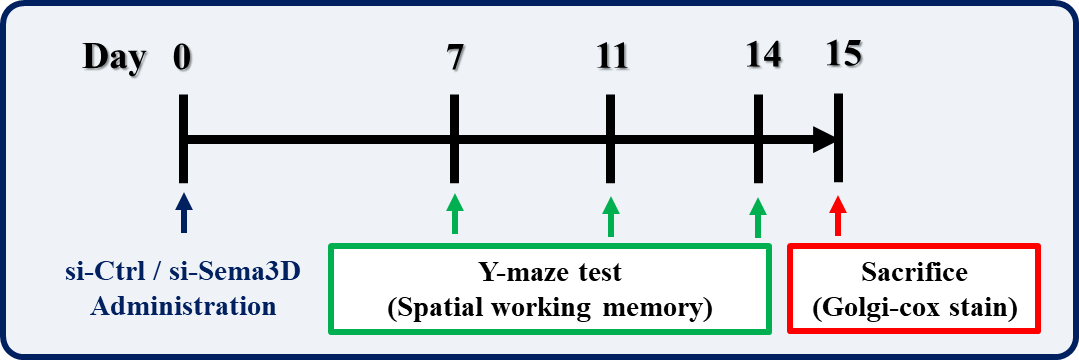
(4B)


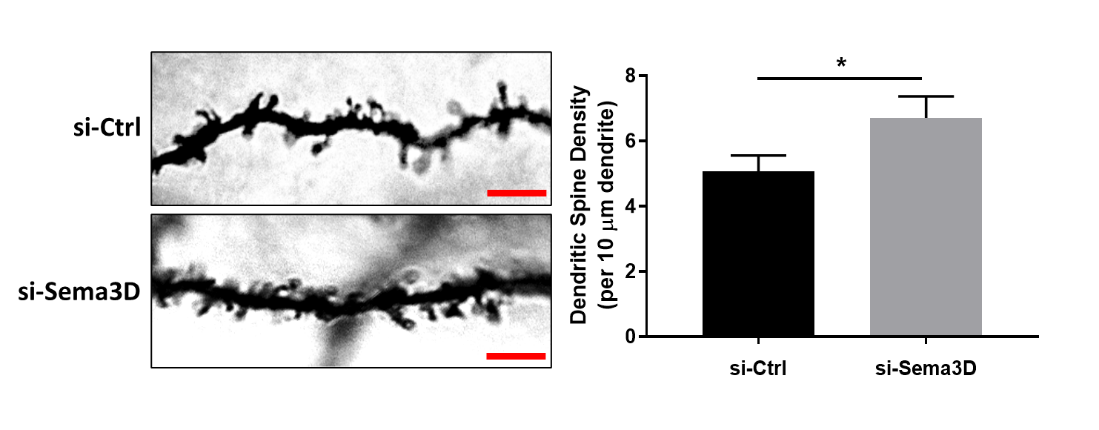
(4C)


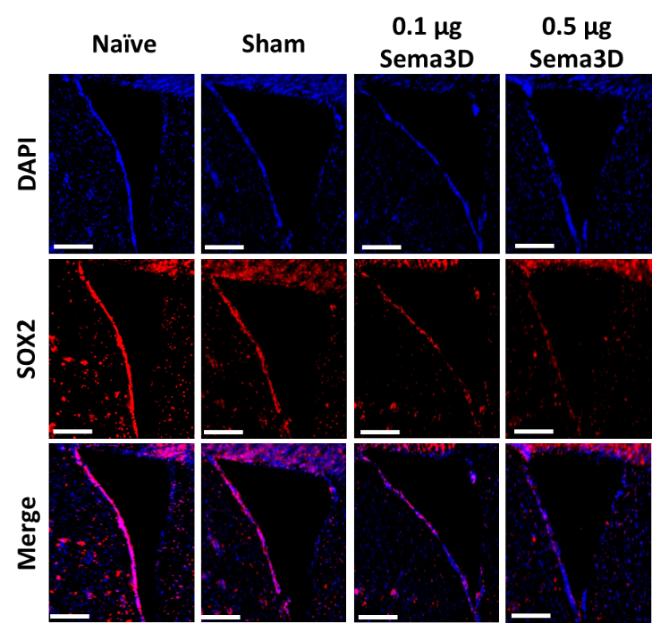
(4D)


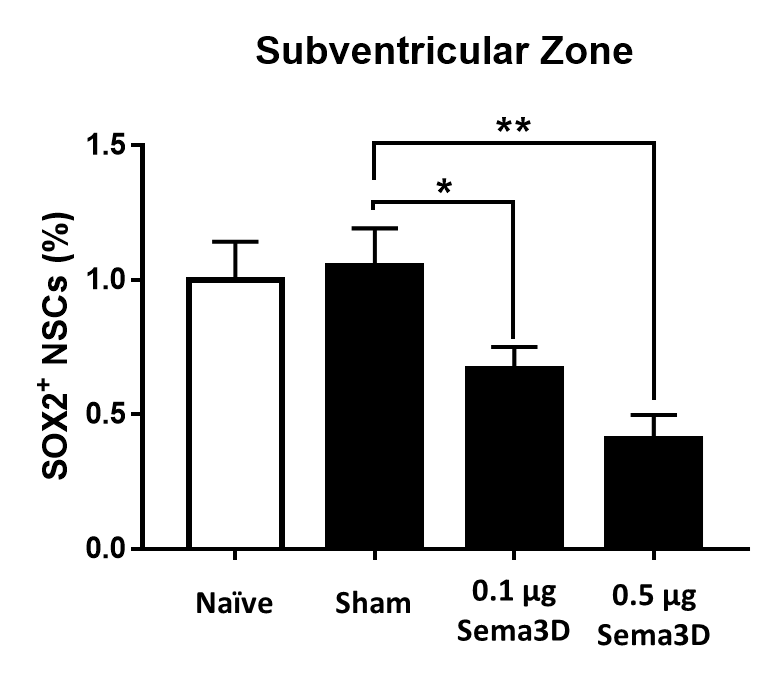


(4E)


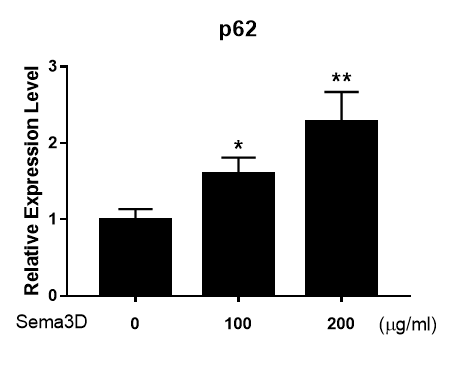

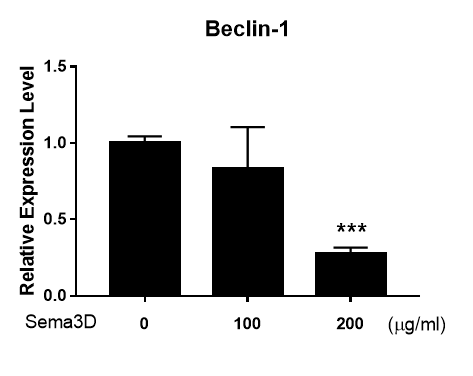

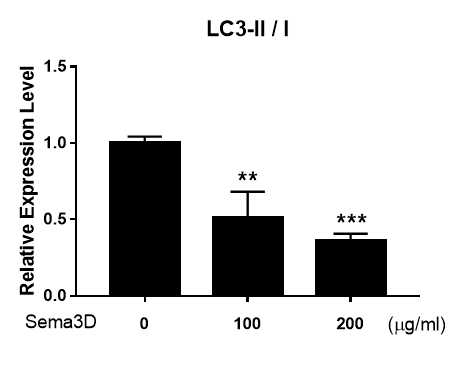

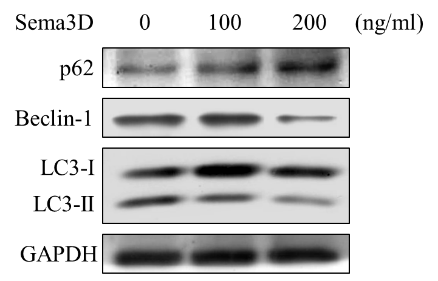


(4F)


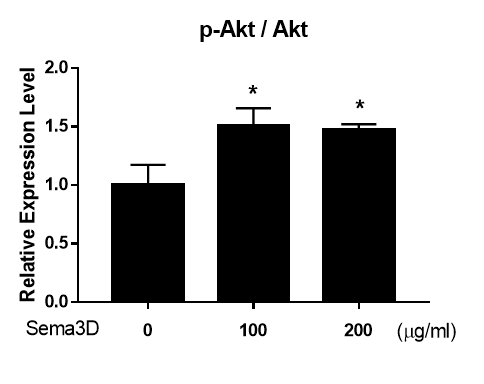

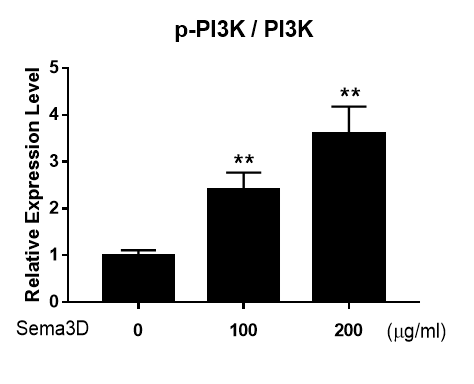

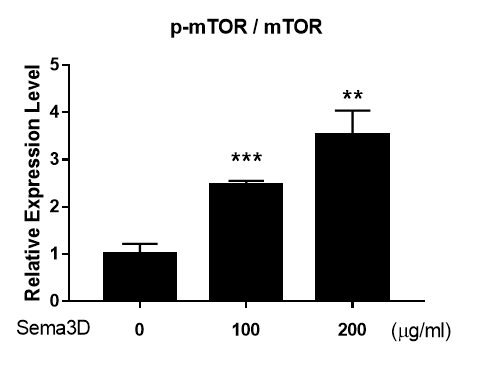

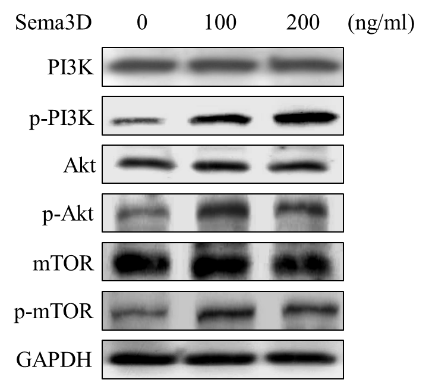


**Supplementary Fig 4. The effect of Sema3D on neural cells and autophagy-associated PI3K/Akt/mTOR pathway**

(A) Representative images of immunofluorescence stain in the hippocampal dentate gyrus (DG) show the efficiency of Sema3D knockdown. siRNA-Sema3D or siRNA-Ctrl was injected to bilateral hippocampi of 12-month-old miR-195a KO mice. Immunofluorescence staining was conducted at 7 day-post siRNA administration (n=3/group). Quantitative data on the right. (B) The Y-maze test measuring spatial working memory was used to assess the effect of siRNA-Ctrl or siRNA-Sema3D injected into miR-195a KO mice aged 12 months. The scheme shows details of siRNA administration to miR-195a KO mice, the days for tests, and brain sample collection. (C) Representative images of dendritic spine density of CA1 pyramidal neurons in siRNA-Sema3D injected mice (n=3 mice/group; 15 neurons/mice). siRNA-Sema3D or siRNA-Ctrl was injected to bilateral hippocampi of 12-month-old miR-195a KO mice that were then sacrificed on day 15-post injection. Scale bar = 5 μm. Quantification of dendritic spine density on the right panel. (D) The NSC population of 4-month-old WT mouse was measured at 72 h-post Sema3D ICV injection. Representative images of SOX2+ (red) NSCs in the subventricular zone (SVZ) of Sema3D-injected mice (left panel). Quantification of SOX2+ cells in SVZ on the right. Magnification: 20X. Scale bar = 200 μm. (n=3/group). Data are presented as mean ± SEM. **p* <0.05; ***p* < 0.01 versus the data on the Sham group. (E) Western blots of autophagy-associated proteins p62, Beclin-1, and LC3-II / I of human neurons at 72 h-post Sema3D treatment. Quantitative western blot data on the right. (F) Western blots for the effect of Sema3D on the phosphorylation of PI3K/Akt/mTOR signaling pathway at 24 h-post Sema3D treatment. Quantitative western blot data on the right. All quantitative data are presented as mean ± SEM from three independent experiments, **p* < 0.05, and ***p* < 0.01.


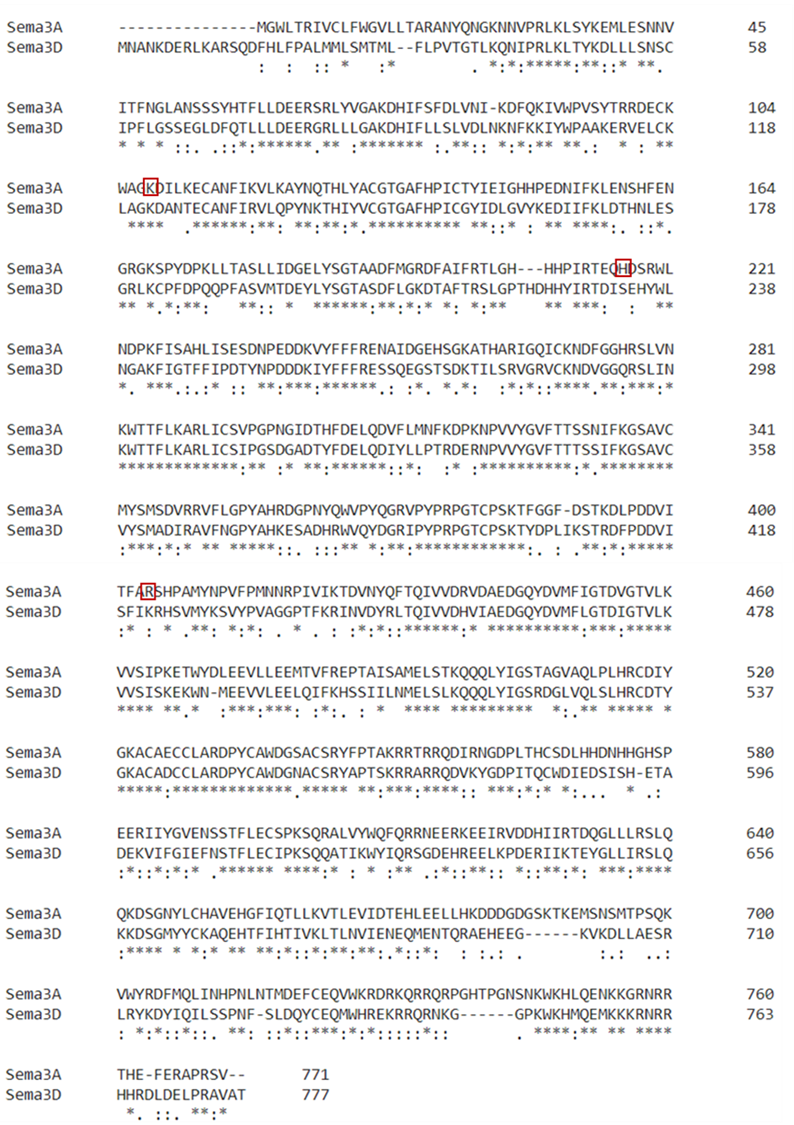
(5A)

(5B)


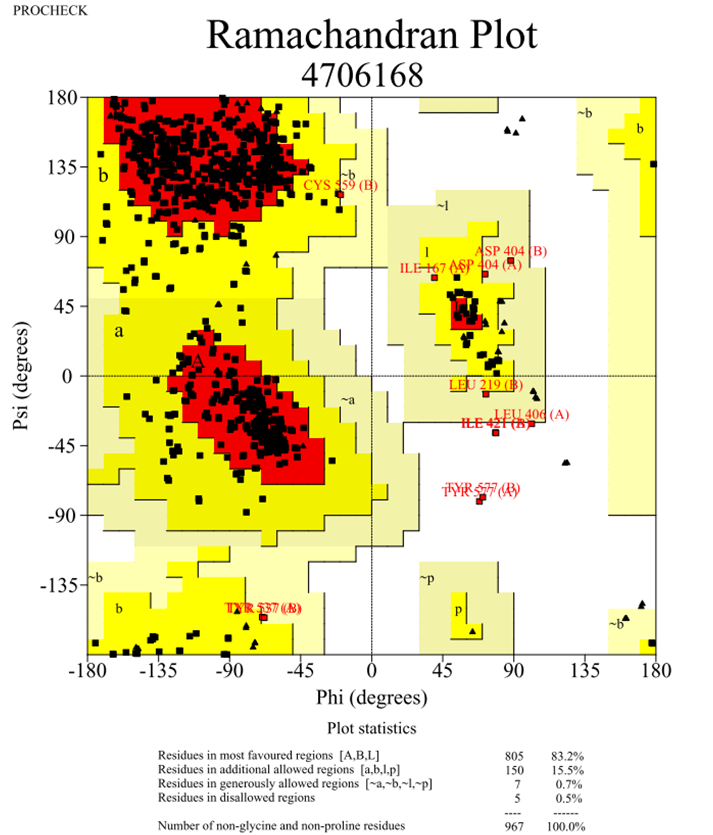


**Supplementary Fig 5. Prediction of Sema3D structure.**

(A) Alignment of amino acid sequences of Sema3A and Sema3D. The complete sequence of *Homo sapien*s Sema3D protein contains 777 amino acids and the molecular weight is 89.65kDa. Sequence identity between *Mus musculus* Sema3A and *Homo sapiens* Sema3D is 60.04%, and the critical residues K108, H216 and R404 in the interface between Sema3A and PlexinA2 are shown in red box. (B) Ramachandran plot of *Homo sapiens* Sema3D model by PROCHECK. The quality of the predicted Sema3D has good integrity according to 83.2% of residues in favorable regions, 15.5% of residues in additional allowed regions, 0.7% of residues in generously allowed regions and 0.5% of residues in disallowed regions.

**Table S1. Results of behavioral tests in miR-195a KO mice, Sema3D-overexpressing mice, and Sema3D siRNA-injected mice.**

Behavior data on miR-195a KO vs. WT mice in different age levels. Young: 3-5 months, Adult: 6 - 12 months, Aged: 15-24 months. The age effects on Sema3D-overexpressing mice and Sema3D siRNA-injected mice were not evaluated. MWM: Morris Water Maze; OFT: Open Field Test. N.S.: no statistical significance.

**Table S2. Available GEO datasets on gene expression in hippocampus, cortex, and cerebellum.**

**Table S3. Stereotactic coordinates for all experiments in the present study.**
